# Thermal Anisotropy Ratio >1000 in Solution-Spun Macroscopic Carbon Nanotube Films

**DOI:** 10.1021/acs.nanolett.6c00663

**Published:** 2026-06-13

**Authors:** Ognyan Stefanov, Beomgyu Choi, Junichiro Shiomi, Geoff Wehmeyer

**Affiliations:** ‡ Department of Mechanical Engineering, 3990 William Marsh Rice University, Houston, Texas 77005, United States; § The Carbon Hub, William Marsh Rice University, Houston, Texas 77005, United States; ⊥ Department of Mechanical Engineering, 13143 The University of Tokyo, 7-3-1 Hongo, Bunkyo-ku, Tokyo 113-8656, Japan; ∥ Institute of Engineering Innovation, 13143 The University of Tokyo, 7-3-1 Hongo, Bunkyo-ku, Tokyo 113-8656, Japan; ¶ The Smalley-Curl Institute, William Marsh Rice University, Houston, Texas 77005, United States

**Keywords:** Carbon nanotube film, Thermal conductivity, Thermal diffusivity, Laser flash method, Thermal anisotropy, Thermal management

## Abstract

Films with large anisotropy ratios (*r*) between the in-plane and cross-plane thermal conductivity (κ) can be used for directional heat spreading in electronics thermal management. Here, we show that commercially available solution-spun carbon nanotube (CNT) films with 20 μm thickness and centimeter-scale lateral dimensions exhibit orthotropic thermal conductivity with the highest reported *r* to date, reaching *r* = 1400 ± 160 at room temperature (*T*). We find *r* using laser flash thermal diffusivity (α) measurements over a *T* range from 198 to 573 K. Dedoping of acid residuals via annealing increases the in-plane-aligned α_*x*_ of dedoped samples by a factor of 2 compared to the doped samples. These dedoped CNT films also display a strong α_*x*_ ∝ *T* ^–1.1^ scaling, indicating that phonon–phonon scattering impacts heat transport along the direction of alignment. Our work motivates further exploration of ultrahigh *r* in macroscopic CNT materials and applications of CNT films for directional heat spreading.

## Graphical abstract

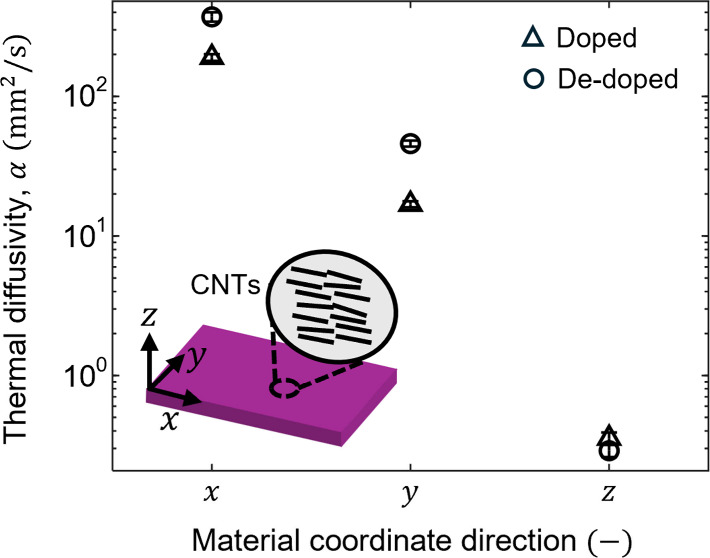

To meet the thermal management needs of chiplet-based heterogeneous integration,^1^ materials with large differences between in-plane and cross-plane thermal conductivities (κ) near room temperature (*T*) can offer directional heat spreading.^2^ Thermal anisotropy allows horizontal heat spreading from heat-generating elements to lateral heat sinks, while preventing vertical coupling from the heat source to sensitive elements that have lower *T* ratings.^3,4^ Currently, layered materials (e.g., van der Waals materials with 2D atomic structures) are the most suitable choice for directional heat spreaders due to their high anisotropy ratios (*r* ≡ κ_high_/κ_low_) at room temperature. For example, highly oriented pyrolytic graphite (HOPG) pairs an ultrahigh in-plane κ of 1870 W/m·K with a lower cross-plane κ of 7.4 W/m·K, leading to *r* = 253.^5^ MoS_2_ thin films without interlayer registry display a moderate in-plane κ of 50 W/m·K and an ultralow cross-plane κ of 0.057 W/m·K, leading to record values of *r* = 877.^6^

Although layered materials currently display the highest *r*, chain-like anisotropic materials made from constituent elements with 1D atomic structure offer an alternate route for directional heat spreading along one preferential in-plane direction. Polymers can display high *r* if the molecular chains are aligned via stretching,^7^ and polyethylene films display *r* ≈ 100 at room temperature.^8^ However, stretched polymers have only moderately high along-alignment κ,^7^ with polyethylene nanofibers reaching 104 W/m·K^9^ and polyethylene films achieving 65 W/m·K.^10^ In contrast, individual carbon nanotubes (CNTs) have much higher measured axial κ (at least >1000 W/m·K),^11,12^ and many CNT materials display notable thermal anisotropy.^13–23^ The upper bound of *r* in CNT materials is difficult to determine,^24–26^ but measurements on nanoscale CNT bundles have shown *r* ≫ 100 at both 1000 ^27^ and 200 °C.^28^

The current room-temperature experimental record in macroscopic CNT materials is *r* ≈ 500, which was achieved in a thin film by aligning single-wall CNTs via vacuum filtration.^13^ These single-wall CNT films show quite low κ in the cross-plane direction perpendicular to alignment, achieving values of κ = 0.085 ^13^ or 0.13 W/m·K^29^ in different demonstrations. Although we are not aware of first-principles calculations of the κ perpendicular to alignment in fully densified close-packed CNT networks, these low measured κ values could be due to a combination of intrinsically low κ in the radial direction of packed CNTs due to weak intertube bonding, interfacial resistances at the junctions between CNT bundles,^30,31^ and/or the effects of incomplete densification. The along-alignment κ in the vacuum filtration study of 43 W/m·K was limited by the 0.2 μm CNT length.^13^ Although it is challenging to align long CNTs via vacuum filtration, few-wall purified CNTs with much longer tube lengths (∼10 μm) can be aligned using solution-spinning methods.^32–34^ The resulting CNT assemblies have measured along-alignment κ as high as 380 W/m·K for as-spun fibers^34,35^ and 777 W/m·K for postprocessed fibers.^36^ Although the high degree of alignment and long CNT lengths are promising to achieve high *r*, the thermal anisotropy of these solution-spun CNT assemblies has not been reported to date.

Here, we find that macroscopic solution-spun CNT films exhibit the highest measured *r* (1400 ± 160) at room temperature for any material class. We measure the anisotropic thermal diffusivity (α = κ/*C*, where *C* is the heat capacity per unit volume) in the in-plane-aligned (*x*), in-plane perpendicular (*y*), and cross-plane (*z*) directions using a laser flash method with relatively short (down to 20 μs) heating pulse times. This anisotropy arises from the combination of a low α_*z*_ (0.25 ± 0.03 mm^2^/s, corresponding to κ_low_ = 0.19 ± 0.06 W/m·K) and a high α_*x*_ (360 ± 18 mm^2^/s, corresponding to κ_high_ = 270 ± 70 W/m·K) at 293 K for the dedoped sample. α_*x*_ decreases with increasing *T* over a range from 195 to 573 K due to a combination of the *T*-dependent phonon occupation statistics (which increase *C* with increasing *T*) and the *T*-dependent phonon–phonon scattering (which can decrease κ_*x*_ with increasing *T*). The in-plane perpendicular direction displays an α_*y*_ that is 2 orders of magnitude larger than α_*z*_ (46 ± 3 vs 0.25 ± 0.03 mm^2^/s at 298 K in the dedoped condition), which we attribute to partial CNT orientation along the *y* direction and lack of CNT orientation along the *z* direction. The highest *r* values correspond to films that have undergone moderate *T* annealing to remove residual acid from the spinning process and dedope the films. The dedoped films have larger α_*x*_ and stronger α_*x*_(*T*) scaling due to the reduced importance of phonon-impurity scattering compared to Umklapp scattering. Thus, our work demonstrates ultrahigh *r* in macroscopic CNT assemblies and motivates further investigation of multidirectional thermal transport for heat spreading applications in electronics thermal management.

Figure 1a shows an optical image of the CNT film commercially produced by DexMat (Houston, TX, USA). The CNT films have thicknesses *d* that range from 14 to 23 μm, widths of 1–2 cm, and long film lengths (up to >10 cm). Figure 1a also defines a material coordinate system with *x* along the CNT alignment direction, *y* in-plane perpendicular to the alignment, and *z* cross-plane perpendicular to the film surface. The CNT film is produced via a solution-spinning process (Figure 1b) in which commercially available purified few-wall CNTs are dissolved in chlorosulfonic acid (CSA), filtered to form a liquid crystalline spinning dope, extruded through a slot-shaped spinneret into a coagulant, and collected on a drum.^34,37^ Transmission electron microscopy (TEM) characterization of these CNTs (Meijo Nano Carbon eDIPS) indicates a mean diameter of 1.9 ± 0.5 nm and a mean wall count of 1.9 ± 0.6.^38^ Rheological measurements of these CNTs in solution^39^ are used to obtain viscosity-averaged CNT molecular aspect ratios of 5600, corresponding to a viscosity-averaged mean CNT length near 10 μm.^38^ Raman measurements on the CNT films shown in Figure S1 show the expected low-wavevector radial breathing modes, a large G peak from the sp^2^ carbon bonding, and a limited D peak due to disorder.^40^ Resonant Raman conditions are observed at multiple excitation wavelengths due to the heterogeneous range of CNT diameters in the sample.^41^ The large Raman G/D ratios of >50 at 633 nm excitation wavelength (Figure S1) confirm the high crystallinity of the constituent CNTs.

**Figure 1:**
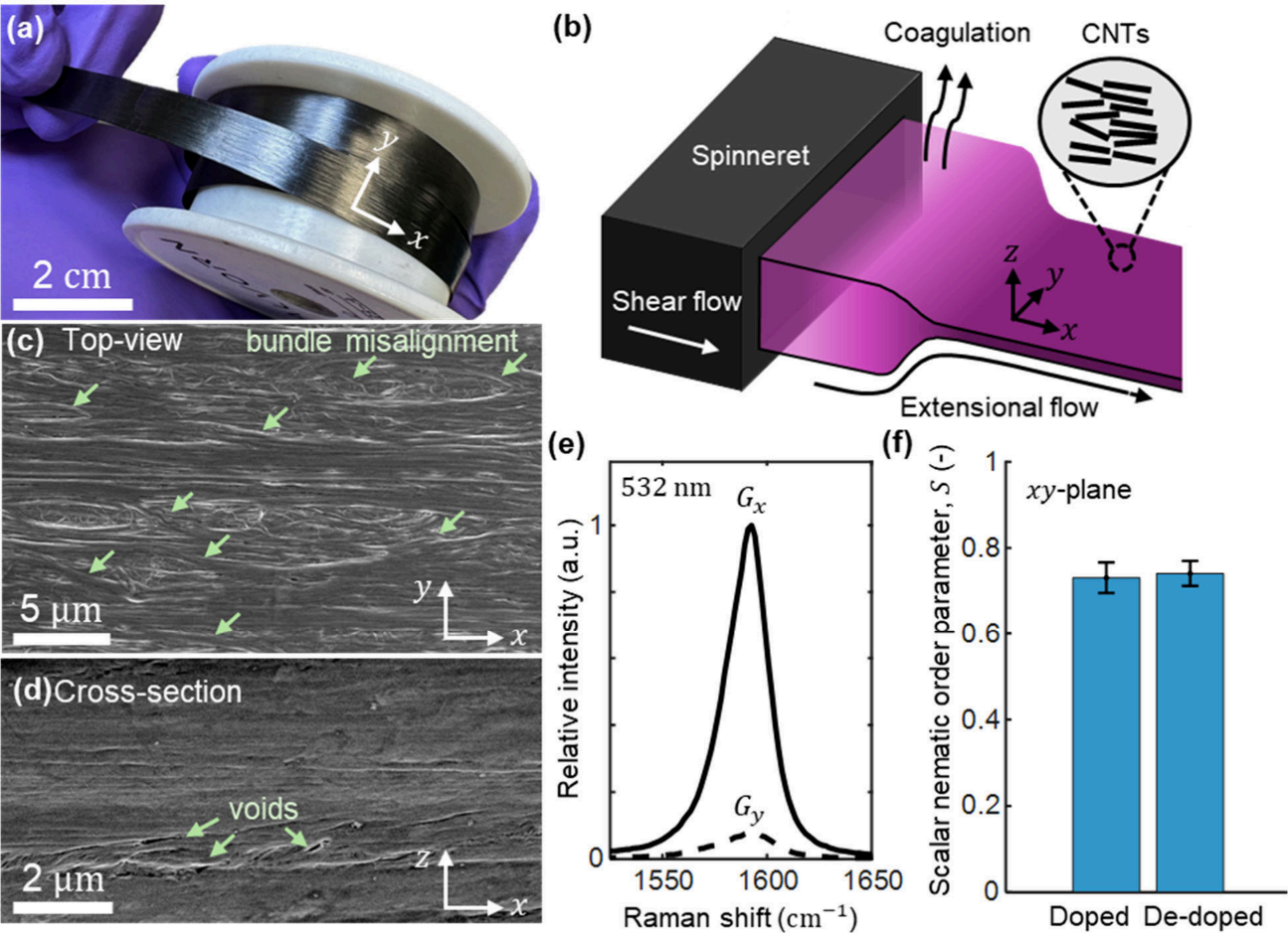
Characterization of the structural hierarchy and anisotropy in solution-spun CNT films. (a) Top-down optical image of CNT film on spool. (b) Schematic of the wet-spinning process and resulting CNT alignment along the *x* direction. (c) SEM top-view and (d) 52° projection of the *xz* plane for dedoped film after razor blade cut. Arrows label regions of observed bundle misalignment in part c and void regions in part d. (e) Polarized Raman spectra acquired using a 532 nm excitation wavelength on the film surface in the *xy* plane, used to extract (f) the scalar nematic order parameter (*S*) of doped and dedoped CNT films. These polarized Raman measurements show that *S* is relatively large and is unaffected by the <900 °C annealing temperature used to dedope CSA residuals.

The resulting CNT materials contain residual CSA derivatives that act as p-type dopants,^34,41^ and moderate-*T* annealing can remove these CSA derivatives.^34,41^ We dedoped the CNT film by annealing segments of film in an inert gas environment (nitrogen or argon) at 800–900 °C for 4–6 h. These annealing conditions reduced the *x* component of the electrical conductivity (σ_*x*_) from 5.4 ± 0.3 to 1.5 ± 0.08 MS/m, which is consistent with previous reports on annealed CNT fibers.^34,41^ Dedoping also substantially reduces the CNT film density from 1300 to 870 kg/m^3^, due to the removal of CSA residuals. This annealed CNT film density is substantially lower than the density of 1710 kg/m^3^ observed in dedoped solution spun CNT fibers made from similar CNTs,^42^ indicating that the CNT film is not fully densified. Prior work on these CNT fiber structures also indicates that dedoping may assist in reducing intertube spacing within a CNT bundle.^42^ Throughout this manuscript, we refer to as-received CNT films as “doped” and annealed CNT films as “de-doped”.

Parts c and d of Figure 1 show top-view (*xy* plane) and cross-sectional (52° projection of the *xz* plane) scanning electron microscopy (SEM) images, which display relatively high bundle alignment along the *x* direction along with notable voids/porosity in these annealed CNT films. Although the overall alignment is along the *x* direction, the top-view SEM shows that some regions have local bundle alignment with components in the *y* direction indicated by the arrows in Figure 1c. The cross-sectional SEM shows limited contrast with no observable bundling and strong orientation along the *x* direction, although some void regions are observed (see arrows in Figure 1d). The films display strong *x*-direction alignment in the *xz* plane because the CNT film shrinks substantially in the *z* direction during the coagulation process, as discussed in Supplementary Note A. We quantify the CNT alignment at the *xy*-plane surface using polarized Raman spectroscopy with a 532 nm excitation wavelength (Figure 1e) and obtain a G-peak intensity ratio of G_*x*_/G_*y*_ = 11 for measurements where the incident and scattering polarizations are parallel (G_*x*_) and perpendicular (G_*y*_) to the grain. We calculated the 2D scalar nematic order parameter (*S*) from the G-peak intensities, including one additional configuration for the incident/scattering laser polarization not shown in Figure 1e.^43^ Figure 1f shows that doped CNT film displays an order parameter of *S* = 0.73 ± 0.04, where the error bar represents standard error due to measurements at multiple locations. The annealing process used to remove the residual acid causes macroscopic sample shrinkage along the width (quantified in Figure S2) but does not have a major influence on the Raman-measured CNT alignment, as the same doped sample has a similar *S* = 0.74 ± 0.03 after dedoping. These order parameters are smaller than that observed in dedoped solution-spun CNT fibers, because these CNT fibers exhibit better densification and alignment along the axial direction compared to the CNT films.^42,44^

We measured the cross-plane thermal diffusivity (α_*z*_, Figure 2a) and in-plane thermal diffusivity parallel to alignment (α_*x*_, Figure 2b) using the laser flash method (NETZSCH Light Flash Apparatus (LFA 467) HyperFlash).^45^ For the cross-plane configuration shown in Figure 2a, a xenon light pulse (red) heats the bottom of the sample while an infrared (IR) sensor detects the IR emission from the top of the sample (green, representing the detection area). The in-plane measurement in Figure 2b utilizes two masks to localize both the *T* measurement and the heating. The top mask had a single central slit (8 mm in *y* by 1 mm in *x*) that allowed the IR detector to probe the upper surface *T* near the central region. The bottom mask had two slits of the same dimensions, positioned symmetrically about the center with a bottom-mask slit-to-slit distance of 2*L*, to allow the pulse to heat the bottom surface. Here, the slit separation distance *L* is the in-plane distance between the center of the top mask’s middle slit and the center of the bottom mask slit.

**Figure 2:**
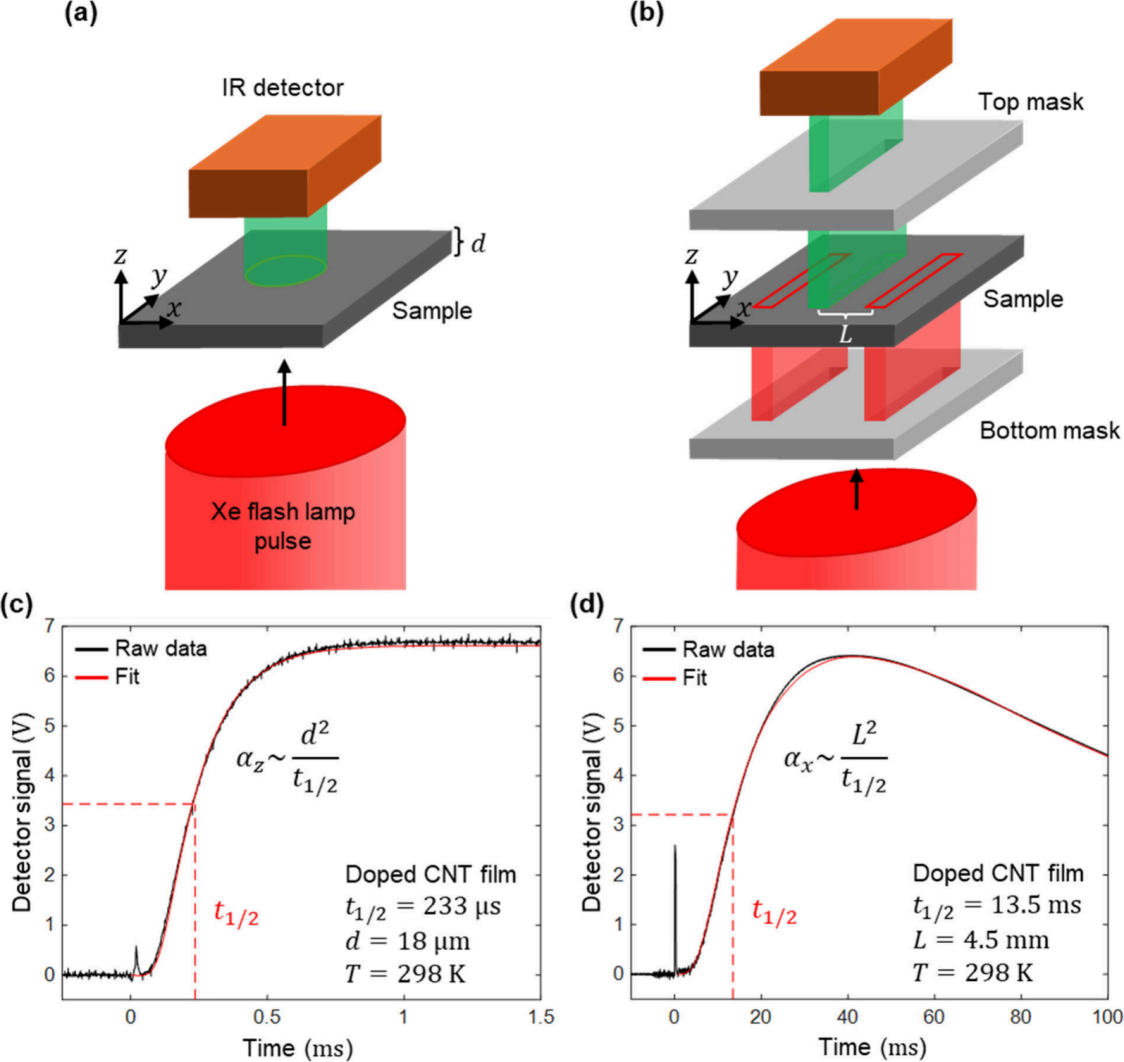
Laser flash configurations and corresponding IR detector signals. (a and b) Schematics of cross-plane and in-plane measurement configurations, respectively. Time-dependent voltage signals from the IR detector for the (c) cross-plane configuration and (d) in-plane configuration, both for a CNT film at room temperature. Black solid lines represent the measured detector signal, while solid red lines correspond to the fitting models used to extract α. Note the shorter time scale for cross-plane measurements in part a.

Parts c and d of Figure 2 show representative measured data from cross-plane and in-plane CNT film measurements, respectively, at 298 K. In the simplest analysis,^45^ the component of α parallel to the *T* gradient is proportional to the square of the characteristic diffusion length (*L* for in-plane or *d* for cross-plane) divided by *t*_1/2_, which is the half-rise time of the detector signal. The cross-plane time scales are much faster than the in-plane time scales because *d* ≪ *L*. For every measurement, we set the xenon lamp pulse width (*t*_pulse_) to no more than one-fifth of *t*_1/2_. The resulting adiabatic temperature rise is typically <10 K (see Supplementary Note B), ensuring operation within the linear response regime. We obtained α by fitting the full time-dependent detector signal with thermal models (curve-fitting method^46^) implemented in the NETZSCH Proteus analysis software including the effects of finite pulse width, finite slit width, heat losses, and baseline offsets. The sharp signals near time *t* = 0 ms are detector noise that is associated with the pulse (either due to electrical noise or direct impingement of reflected lamp pulse energy onto the detector); these sharp nonthermal signals are discarded for the data fitting (red lines). The uncertainty in α was estimated by propagating uncertainties in the characteristic diffusion length through the half-rise time formulation, as described in Supplementary Note B.

We validated the laser flash technique using *T*-dependent in-plane and cross-plane measurements performed on 98-μm-thick HOPG samples obtained from HPMS Graphite and found good agreement with literature values^5^ (Figure S3). We verified that CNT film measurements performed with and without a graphite coating yield similar thermal diffusivity, as shown in Figure S4 and discussed in Supplementary Note B, and that the cross-plane and in-plane measurements yield accurate results for a 320 μm isotropic nickel foil, as shown in Figure S12. We also validated that the short-pulse laser flash technique can be applied to relatively thin films by performing room-temperature cross-plane measurements on a coated 25-μm-thick polyimide film (Figure S4c) and in-plane measurements on a 16-μm-thick HOPG film (Figure S3b), again finding good agreement with literature results.

Figure 3 shows the laser flash measurements for doped (triangles) and dedoped (circles) CNT films over the *T* range from 198 to 573 K. Each color represents a different sample, and the dashed lines denote the power-law fits to each data set. Combining these α measurements with heat capacity measurements in Figure S5 obtained over a smaller *T* range from 215 to 340 K can yield κ­(*T*), which is shown in Figure S6. Figure S7 shows that *C*(*T*) of the CNT samples displays a similar *T* trend as that of graphite over the measured *T* range. We focus our discussion in the main text on the diffusivity results because α is extracted directly from the laser flash measurements, and because we obtain α over a larger *T* range.

**Figure 3:**
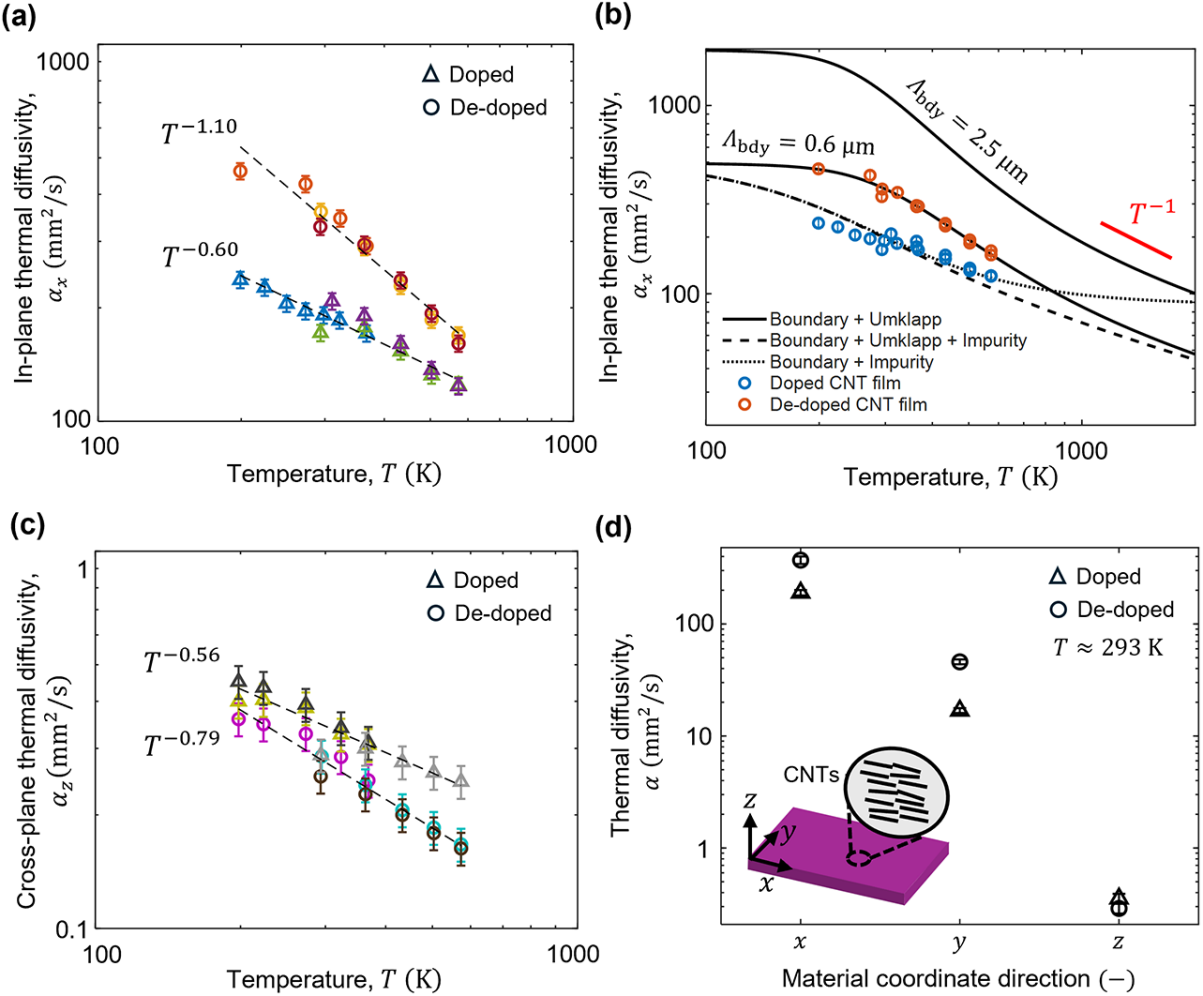
Temperature-dependent thermal diffusivity of solution-spun CNT films. (a) The in-plane thermal diffusivity of doped (triangles) and dedoped (circles) films decrease with increasing *T*. Colors represent measurements on different samples, and dashed lines represent power law fits. Removing the acid residuals in dedoped CNT films increases the in-plane diffusivity and strengthens the temperature trend of α_*x*_(*T*). (b) Fitting a 1D Born–von Karman Slack phonon transport model for α_*x*_(*T*) in packed CNTs to the doped and dedoped data indicates that Umklapp scattering plays an important role in α_*x*_(*T*) of dedoped films and that even higher α_*x*_(*T*) would be possible with larger boundary scattering mean free paths Λ_bdy_. (c) Cross-plane thermal diffusivity of doped (triangles) and dedoped (circles) samples show weaker temperature scaling and 3-order-of-magnitude reduction of α_*z*_ compared to α_*x*_. (d) The CNT film thermal diffusivity tensor is orthotropic due to the partial CNT orientation in the *y* direction and minimal orientation in the *z* direction. Error bars in all experimental results represent the standard error with a 68% confidence interval, dominated by uncertainties in the characteristic diffusion length (*d* or *L*).

First focusing on the in-plane along-alignment α_*x*_ measurements in Figure 3a, α_*x*_ of doped CNT films decrease from 240 ± 12 to 120 ± 6 mm^2^/s with increasing *T* from 198 to 573 K, displaying a best-fit scaling dependence of *T* ^–0.60±0.05^. The dedoped CNT films exhibit systematically higher values of α_*x*_ over the same *T* range (460 ± 23 to 160 ± 8 mm^2^/s), with a stronger temperature scaling (*T* ^–1.1±0.07^) relative to the doped films. The 80% improvement in α_*x*_ upon dedoping and stronger *T* scaling in the dedoped samples is qualitatively consistent with previously observed 60% enhancement and stronger *T* scaling in the axial κ of annealed solution-spun CNT fibers, which was attributed to a reduction in phonon impurity scattering upon removal of the CSA residuals.^34^

To quantify this effect, we use phonon thermal modeling described in Supplementary Note C and Figure S10 to explore the along-alignment transport mechanisms. Using the Wiedemann–Franz law along with our measured σ_*x*_ values of the CNT films shown in Table S1 indicates that phonons are the dominant heat carriers in the *x*-direction, for both doped and dedoped samples. Following standard approaches,^47^ the phonon model considers a 1D sine-type Born–von Karman phonon dispersion relation and includes boundary, impurity, and Umklapp scattering mechanisms. Figure 3b shows that fitting this phonon transport model to the data indicates that impurity scattering and boundary/interfacial scattering are the strongest scattering mechanisms for α_*x*_ of the doped samples. A model that excludes Umklapp scattering (dotted line) has similar α_*x*_(*T*) for the doped sample as the model that includes Umklapp scattering (dashed line). In contrast, we fit the dedoped sample α_*x*_ (*T*) with a combination of Umklapp scattering and boundary scattering, and find good agreement with the fit for a boundary scattering mean free path of Λ_bdy_ = 0.6 μm. The model predicts that even higher CNT α_*x*_ with stronger *T* scaling (∼*T* ^–2.0^) could be observed for larger Λ_bdy_, which is qualitatively consistent with the strong α_*x*_(*T*) scaling of *T* ^–2.2^ for HOPG as shown in Figure S3.

Now turning to the cross-plane measurements, Figure 3c shows that α_*z*_ is much smaller than α_*x*_, reflecting the underlying anisotropy in the CNT alignment. The α_*z*_ of doped CNT films ranges from 0.45 ± 0.05 to 0.25 ± 0.02 mm^2^/s, and has a relatively weak temperature scaling of *T* ^–0.56±0.06^. The dedoped films have α_*z*_ that are lower than the doped films, with values ranging from 0.36 ± 0.04 to 0.17 ± 0.02 mm^2^/s with a slightly stronger temperature dependence of *T* ^–0.79±0.05^. The 3-order-of-magnitude reduction in α_*z*_ compared to α_*x*_ indicates that there is minimal CNT bundle orientation along the *z*-direction. At room temperature, these double-wall CNT dedoped films have κ_*z*_ = 0.19 ± 0.06 W/m·K, which is larger than κ_*z*_ in single-wall CNT films (0.083 ^13^ to 0.13 W/m·K^29^ for CNT diameters of 1.4 nm) and 1 order of magnitude smaller than κ_*z*_ for multiwall CNT films (4 ^16^ and 6.4 W/m·K,^48^ for CNT diameters ranging from 8 to 10–20 nm, respectively) or for HOPG (7.4 W/m·K^5^). Films made from few-wall CNTs have higher CNT–CNT interface densities in the cross-plane direction than films made from multiwall CNTs, which could lead to lower phonon mean free paths and κ_*z*_. To confirm that the cross-plane transport is insensitive to the sample porosity, we performed measurements on CNT films that were mechanically compressed in the *z* direction to achieve 20% higher density (1040 kg/m^3^ for dedoped samples) and found similar κ_*z*_ values of 0.18 ± 0.06 W/m·K, as shown in Figure S13. This finding of low κ_*z*_ for single- and double-wall CNT films compared to multiwall CNT films and HOPG motivates further computational investigation of wall-number-dependent cross-plane heat conduction in CNT films.

Figure 3d showcases the orthotropic thermal conductivity in the doped and dedoped CNT films at *T* = 293 K. We measured the in-plane *y*-direction α_*y*_ near room temperature, as described in Supplementary Note B and shown in Figure S9, and found that α_*y*_ is 1 order of magnitude smaller than α_*x*_ but 2 orders of magnitude larger than α_*z*_. The two perpendicular directions *y* and *z* are inequivalent because CNT bundles are partially oriented along the *y* direction (as observed in the SEM images in Figures 1c and S8 and quantified by the Raman order parameter in Figure 1e), but display almost no orientation along the *z* direction (as observed in the cross-sectional SEM image in Figure 1d). Due to the large intrinsic anisotropy in the phonon dispersion relations of CNTs, heat flows more readily along the axial direction of the CNT bundle than in the radial direction. Therefore, we expect that α_*y*_ is highly sensitive to the bundle misalignment along *y* in the *xy* plane, because transport is dominated by the diffusion of heat along the axial CNT orientation. In contrast, the *z*-direction shrinkage during coagulation ensures minimal bundle alignment along *z* in the *xz* plane, meaning that transport is dominated by the radial coupling between CNTs with no axial contribution. The doping trends in Figure 3c are also consistent because dedoping increases α_*y*_ and α_*x*_ (both of which depend on axial transport in a bundle) but decreases α_*z*_ (which is more sensitive to the radial CNT transport).

One reason why cross-plane thermal transport in thick CNT films has received less attention than in-plane thermal transport is because traditional laser flash methods struggle to measure α_*z*_ in *d* = 20 μm films, since the rise time *t*_1/2_ should be larger than *t*_pulse_ to be sensitive to cross-plane conduction.^49,50^ To examine the accessible experimental regimes, Figure 4a displays α_*z*_ as a function of *d* for CNT films and validation measurements on HOPG and polyimide films. The marker shape denotes the sample type (triangles, doped CNT film; circles, dedoped CNT film; squares, HOPG), and the color represents *T*. Dashed lines correspond to contours of constant rise time *t*_1/2_ = 0.1338 *d* ^2^/α_*z*_.^45^ Figure 4a shows that the CNT and HOPG samples have similar *t*_1/2_ values ranging from 100 to 200 μs. Comparing *t*_1/2_ for our experiments with the “pulse-width limited” region (red, *t*_pulse_ = 20 μs) in Figure 4a shows that the CNT and HOPG measurements are not pulse-width limited. Supporting Information Figure S4d shows the measured time response in a pulse-limited laser flash measurement for a 16-μm-thick HOPG film in the cross-plane configuration (labeled “thin HOPG” in Figure 4a), confirming that the detector would be able to measure much faster rise times than we observed for the CNT films. We also verified that α_*z*_ of the CNT films at a given *T* and doping level does not depend strongly on *d* over the range considered here (compare data in Figure 4a measured over *d* from 14 to 23 μm, the full range of available thicknesses).

**Figure 4:**
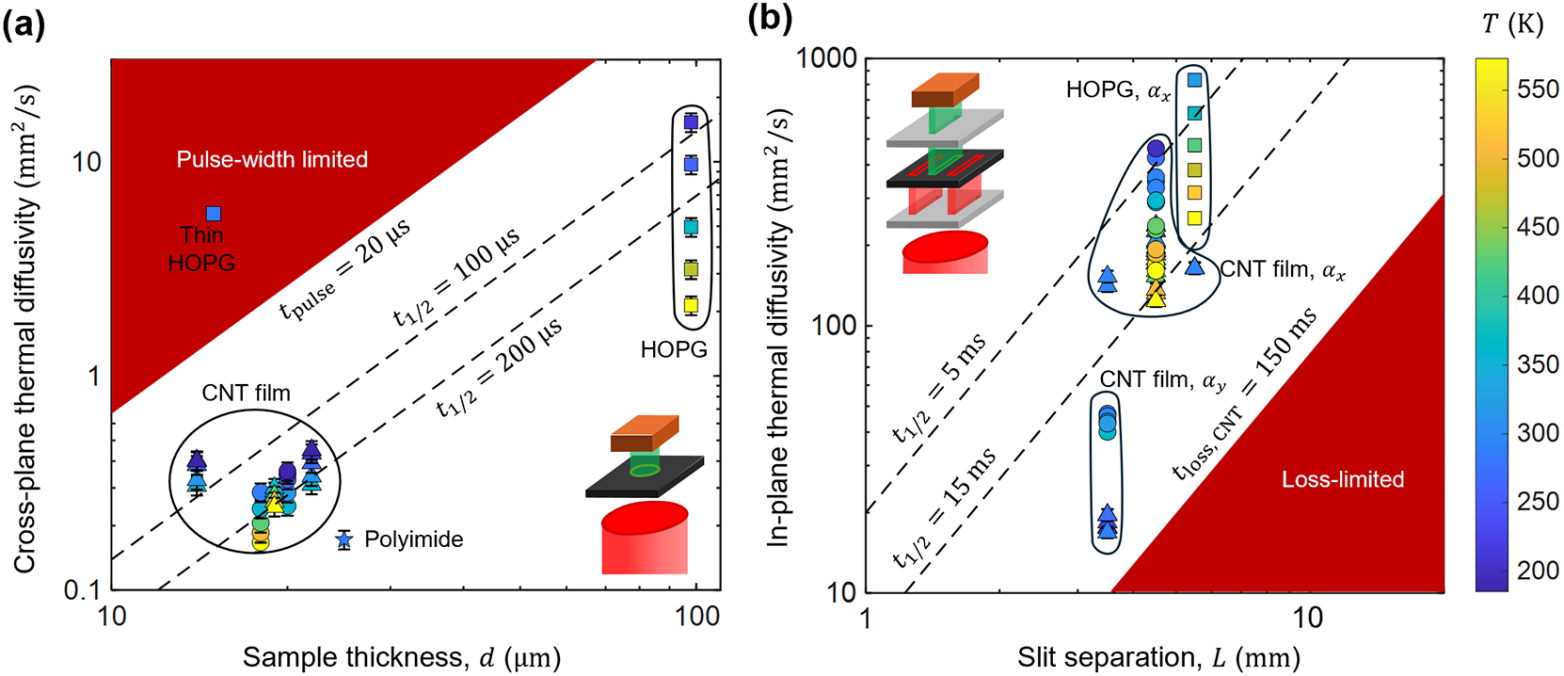
Comparison of relevant heat-transfer time scales and laser flash limitations as a function of the characteristic length for (a) cross-plane and (b) in-plane measurement configurations. Color represents the sample temperature and marker shapes denote the sample type: doped CNT film (triangles), dedoped CNT film (circles), thick and thin validation HOPG films (squares), and validation polyimide film (stars). The thick HOPG and CNT films have similar heat-transfer time scales in both the cross-plane and in-plane along-alignment configurations, and these time scales are appropriate for the laser flash methodologies pursued here. Error bars representing propagated uncertainty are smaller than the data points in most cases.

For the in-plane measurements, the rise times are slower and the primary limitation is the time scale associated with parasitic heat losses (*t*_loss_), which we estimate to be ∼150 ms for our CNT films (Supplementary Note D). Figure 4b compares the in-plane rise time *t*_1/2_ = 0.1*L* ^2^/α_*x*_ to *t*_loss_ for all in-plane measurements by plotting α_*x*_ as a function of *L*. The *x*-direction CNT and HOPG samples have similar half-rise times of 5–15 ms, and in all cases, *t*_1/2_ is smaller than *t*_loss_, as desired. Figure 4b also plots five additional data points from a validation study showing that the extracted α_*x*_ of the CNT films does not depend on *L* over the range 3.5–5.5 mm, further validating the measurement (see also Figure S11).

The thermal anisotropy ratio (*r* = α_*x*_/α_*z*_) of the solution-spun dedoped CNT films at 293 K is 1400 ± 160, which is the highest measured *r* to date (Figure 5). In Figure 5, the marker color indicates whether the material has a chain-like (red) or layered (blue) atomic structure, and the shape of each marker describes the macroscopic material morphology. The gray dashed lines represent contours of constant *r*. We convert our α measurements to κ measurements by using differential scanning calorimetry and mass measurements to determine that *C* = 0.76 ± 0.20 MJ/m^3^K for the dedoped CNT film at room temperature (see Supplementary Note F and Figure S7). The solution-spun CNT films are the only material that exceeds *r* = 1000, due to a combination of a relatively large κ_*x*_ (270 ± 70 W/m·K) and a relatively small κ_*z*_ (0.19 ± 0.06 W/m·K). Other extreme anisotropy materials such as HOPG (*r* = 253),^5^ vacuum-filtrated single-wall CNT film (*r* = 500),^13^ and MoS_2_ films without interlayer registry (*r* = 877)^6^ have higher κ_high_ or lower κ_low_ than solution-spun CNT films, meaning that solution-spun CNT films are likely of interest for applications where maximizing *r* is essential (rather than maximizing κ_high_ or minimizing κ_low_). Anisotropic heat spreading can also be observed in composite structures with two isotropic materials of different κ, although the modeling in Supplementary Note E shows that a simple polymer–metal foil composite system has smaller heat spreading anisotropy than the CNT film.

**Figure 5:**
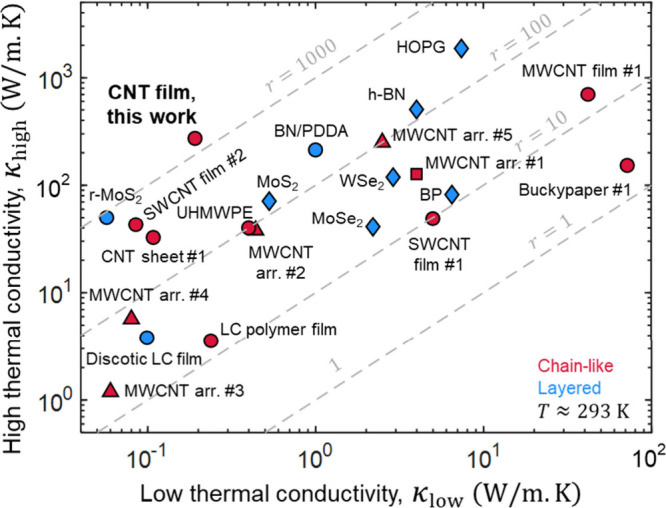
Dedoped solution-spun CNT film (red circle) with the highest reported thermal anisotropy ratio (*r*) near room temperature. Marker color indicates whether the material has a chain-like (red) or layered (blue) atomic structure, and the marker shape describes the macroscopic material morphology: film (circles),^6,8,13–15,21,53–56^ horizontal array (squares),^16^ vertical array (triangles),^17–20^ and bulk/crystal (diamonds).^5,57–60^ Dashed lines represent contours of constant *r*.

In conclusion, we show that dedoped macroscopic solution-spun CNT films can display orthotropic thermal transport with ultrahigh thermal anisotropy between the cross-plane- and in-plane-aligned directions. The dedoped solution spun CNT films have a room-temperature along-alignment κ_*x*_ of that is a factor of ∼6 times larger than that observed in vacuum-filtration-aligned CNT films^13^ due to the longer CNTs used in the solution spinning approach (∼10 μm) vs the vacuum filtration approach (∼0.2 μm), emphasizing the important role of nanotube length in achieving high thermal anisotropy. The dedoped samples display a relatively strong α_*x*_(*T*) trend of *T* ^–1.1^, indicating that phonon–phonon scattering mechanisms combined with residual boundary scattering determine the along-alignment thermal transport in these CNT films. The 2-order-of-magnitude difference between the in-plane α_*y*_ and cross-plane α_*z*_ emphasizes the dominant role of CNT misalignment in interpreting transport perpendicular to global CNT alignment. The κ_*z*_ for this work’s double-wall CNT films is larger than those found in single-wall CNT studies^13,29^ but much lower than those found for large-diameter multiwall CNTs^16,48^ or graphite,^5^ indicating that a higher density of CNT-CNT interfaces impedes cross-plane phonon transport in films made from few-wall CNTs.

Although the reported *r* values of 1400 ± 160 are the highest to date, it may be possible to enhance *r* further. Comparing this work’s solution-spun CNT films to other high-performing CNT film-like assemblies (Table 1) shows that films fabricated via direct spinning^21,23^ and array spinning^22^ have been measured with along-alignment κ ≥ 700 W/m·K at room temperature, which is substantially larger than the current work’s 270 ± 70 W/m·K. The cross-plane κ in these systems has not been reported but could be quite low. Table 1 shows that there also is a strong correlation between along-alignment κ and mass density, because better-aligned samples often have higher density. Further alignment of the solution-spun CNT films could lead to enhanced κ_*x*_ and larger *r*, along with potentially higher strength and σ_*x*_.^37,51,52^ Better alignment along *x* would also lead to much lower κ_*y*_ because the current *y*-direction transport is dominated by the axial conduction along *y*-aligned CNTs. The low κ_*z*_ found here also motivates future measurements and first-principles modeling to evaluate the CNT wall-number-dependent cross-plane phonon transport in CNT films and explore further means for κ_*z*_ reduction in macroscopic films. Thus, high-performance solution-spun CNT films could be promising candidates for directional heat spreading and thermal management of vertically integrated electronics.

**Table 1:** Room Temperature Property Comparison of Horizontally Aligned CNT Films

material	fabrication method	mass density (kg/m^3^)	high in-plane thermal conductivity (W/m·K)	low in-plane thermal conductivity (W/m·K)	cross-plane thermal conductivity (W/m·K)	ref
CNT sheet #1	direct spinning	330	33	28	0.11	^56^
SWCNT film #1	vacuum filtration		43		0.085	^13^
SWCNT film #2	magnetic filter deposition	900	49	5		^14^
MWCNT sheet #1	array spinning		70	8.6		^61^
MWCNT film #3	array spinning and stacking	220	91	6.7	6.4	^48^
MWCNT hor. array #1	shear pressing		127	42	4	^16^
Buckypaper #1	domino pushing	620	153	72		^15^
CNT film	solution spinning	870	270	35	0.19	this work
MWCNT film #1	direct spinning	1590	700	42		^21^
MWCNT film #2	direct spinning	1590	759			^23^
Buckypaper #2	array spinning	1390	766			^22^

## Supplementary Material



## Notes

Additional experimental details and measurements, including information on the CNT film solution-spinning process, laser flash measurements, Raman characterization, validation of the laser flash method on HOPG, CNT, nickel, and polyimide film samples, thermal modeling of losses in the in-plane laser flash measurements, temperature-dependent CNT film heat capacity measurements and comparison with carbon allotropes, phonon thermal transport modeling, and electrical conductivity measurements (PDF)
