# Supplementary material for: Thermal Anisotropy Ratio >1000 in Solution-Spun Macroscopic Carbon Nanotube Films
Source: Nano Lett. 2026 Jun 13;26(25):8082–90. doi: 10.1021/acs.nanolett.6c00663 (PMC13329987; doi:10.1021/acs.nanolett.6c00663)
Supplement: Supplementary file 1 [file nl6c00663_si_001.pdf]

Supporting Information for Thermal Anisotropy Ratio > 1,000 in Solution-Spun Macroscopic Carbon Nanotube Films

Ognyan Stefanov[a), b), #], Beomgyu Choi[c), #], Junichiro Shiomi[c), d)+], Geoff Wehmeyer[a), b), e)+]

a) Department of Mechanical Engineering, William Marsh Rice University, Houston TX, 77005, United States.

b) The Carbon Hub, William Marsh Rice University, Houston TX, 77005, United States.

c) Department of Mechanical Engineering, The University of Tokyo, 7-3-1, Hongo, Bunkyo-ku, Tokyo 113-8656, Japan.

d) Institute of Engineering Innovation, The University of Tokyo, 7-3-1, Hongo, Bunkyo-ku, Tokyo, 113-8656, Japan.

e) The Smalley-Curl Institute, William Marsh Rice University, Houston TX, 77005, United States.

+corresponding authors: gpw1@rice.edu, shiomi@photon.t.u-tokyo.ac.jp

[#]These authors contributed equally

Supporting Information Contents

Supplementary Note A: CNT film solution spinning and annealing process

Supplementary Note B: Laser flash measurements

Supplementary Note C: Analytical model of along-alignment thermal diffusivity for a hexagonal arrangement of CNTs using a 1D Born-von Karman dispersion relation

Supplementary Note D: Characteristic time scale associated with parasitic heat losses for the in-plane laser flash configuration

Supplementary Note E: Heat spreading anisotropy of a simple polymer-metal foil composite

Supplementary Note F: Specific heat capacity measurements of CNT films using differential scanning calorimetry

Supplementary Note G: In-plane electrical resistance measurements on CNT films

Supplementary Note H: Mechanical compression of CNT films

Figure S1. Normalized Raman spectra of doped and de-doped CNT films

Figure S2. Optical images of a CNT film before and after annealing

Figure S3. Validation of laser flash method on HOPG films

Figure S4. Additional validations of the laser flash method on thin films

Figure S5. Temperature-dependent specific heat capacity of solution-spun CNT films

Figure S6. Temperature-dependent thermal conductivity and α_y of solution-spun CNT films

Figure S7. Volumetric heat capacity comparison with other carbon allotropes

Figure S8. SEM image of CNT bundle network in a de-doped CNT film

Figure S9. Detector signals vs. time for y-direction laser flash measurements on CNT film

Figure S10. Analytical Born-von Karman model results: volumetric heat capacity and thermal conductivity

Figure S11. Slit separation distance (L) dependent study of α_x in solution-spun CNT films

Figure S12. Validation of laser flash method on isotropic nickel foils

Figure S13. Cross-plane laser flash measurements on compressed CNT films

Table S1. In-plane electrical conductivities of solution-spun CNT film at room temperature

A. CNT film solution spinning and annealing process

This section describes the production and post-processing of the CNT films, with the goal of explaining the observed structure of the film and orthotropic properties. The schematic in Figure 1b illustrates that as the liquid-crystalline solution is extruded through the spinneret, shear stresses along the inner walls promote reorientation and alignment of the CNT domains. Upon exiting the spinneret into the coagulation bath, stretching during drawing further enhances alignment. Simultaneously, the rapid diffusion of CSA out of the dope and into the coagulant due to solubility differences densifies the structure by improving CNT packing. The combined effects of extensional flow and coagulation lead to pronounced anisotropic shrinkage during film coagulation: the film thickness d (z-direction) decreases by roughly two orders of magnitude during the coagulation, whereas the width (y-direction) contracts by less than 50%. The shear and extensional flow lead to preferential CNT alignment as seen in the xy-plane top-view scanning electron microscopy (SEM) micrograph of Figure 1c. CNT bundles form a well-aligned network along x, with some components of the CNT bundle axis oriented in the y-direction (due to the minimal shrinkage along the y-direction during coagulation). SEM imaging of the xz-plane at a $52°$ projection (Figure 1d) shows a high degree of CNT packing and near-complete x-direction alignment, a direct consequence of the strong z-direction shrinkage during coagulation.

Using an electric furnace (SK-Medical VF-3000A), we annealed few-centimeter long segments of CNT film in an inert gas environment (nitrogen or argon) at $800 - 900$ °C for $4 - 6$ hours. The samples were allowed to cool naturally to room temperature inside the furnace and were then stored under ambient laboratory conditions until measurement. The sample displayed notable shrinkage in the y-direction upon annealing; e.g., for the sample shown in Figure S2, the sample width reduced from 8.8 mm to 7.1 mm after annealing. The mass of the sample in Figure S2 also decreased by almost a factor of 2 upon annealing, from 2.23 mg to 1.17 mg, due to removal of CSA residuals. Removing the CSA leads to y-direction shrinkage because the nanotubes can partially fill the spaces that were previously occupied by CSA, enabling denser packing in the y-direction. Because there was limited y-direction shrinkage during the coagulation, the nanotubes are more misaligned in the y-direction than in the z direction, leaving larger void area for the film to shrink along y after the CSA removal. The thickness of the sample is weakly affected by the annealing because the nanotubes were previously compacted in the z-direction during the coagulation process.

B. Laser flash measurements

We performed all laser flash measurements under atmospheric conditions. The total acquisition window for each measurement was set to $20\ t_{1/2}$. After fixing the pulse width, we adjusted the signal gain and xenon flash lamp voltage to produce a peak infrared detector signal of approximately $5 - 7$ V. For in-plane measurements, bottom masks with slit separation lengths (L) of 3.5 mm, 4.5 mm, and 5.5 mm were available. For temperature-dependent measurements, we used $L = 3.5$ mm (α_y) and $L = 4.5$ mm (α_x) for CNT films, and $L = 5.5$ mm for HOPG films. At room temperature, we used all three mask lengths for the CNT film to perform the L-dependent studies shown in Figure S11. We controlled the temperature of the sample holder by using the instrument heaters coupled to a liquid-nitrogen feedthrough. We collected data over the range of -75 °C to 300 °C using step sizes of $25 - 50$ °C. We cut the sample into a square with side lengths of $8 - 10$ mm, which is large compared to the pulse irradiation diameter of 3.7 mm and enables one-dimensional heat transfer in the z-direction. For the in-plane configuration shown in Figure 2b, we cut the sample into a 9 mm by 25 mm strip with the longer dimension running along x. In all cases, the sample only contacts the aluminum masks at the supported ends, ensuring that in-plane conduction along x was the dominant heat transfer mode within the suspended region.

At each temperature, we collected three laser shots and extracted a thermal diffusivity value from each shot. The reported thermal diffusivity at each temperature is the average of the three values. For each cross-plane measurement, we measured the sample thickness after the laser flash analysis using a digital micrometer. The resulting uncertainties in d (± 1 µm) and L (± 0.1 mm) typically dominate over uncertainties associated with thermal model fitting ($< 1\%$) or measurement variability over repeated measurements on the same sample ($\sim 1\%$). For some in-plane measurements (e.g. doped CNT film y-direction and 16 µm HOPG film), where the measurement or fitting uncertainty exceeded the propagated geometric uncertainty, the larger of the two values was used to estimate the uncertainty in α.

For cross-plane measurements, the sample edges were adhered to the grooves of the aluminum holder using carbon tape. For in-plane measurements we secured the sample to the ends of the aluminum mask by adhering its edges with Kapton tape the adhered regions were located far outside the detector area (> 9 mm). The gap distance between the top mask and the top surface of the sample is ~ 0.1 mm, and the gap distance between the bottom mask and the bottom surface of

the sample is $\sim$1.0 mm. Both mask gaps are small compared to the laser-to-sample and sample-to-detector distances, ensuring that the heating and sensing regions are appropriately identified in the thermal model.

The thermal model for the cross-plane laser flash measurement assumes that the laser energy is absorbed only at the heated surface. To validate this assumption for our CNT films, we performed laser flash measurements on CNT films with a graphite spray coating (Black Guard Spray, Fine Chemical Japan) applied on both sides of the sample. Figure S4a shows the detector signal as a function of time for a 20 μm thickness doped CNT film sample with a ~1 μm thickness graphite spray coating in the cross-plane configuration at room temperature. The time response of the signal is quite similar to that observed in our CNT film measurements without coatings (e.g. as shown in Figure 2c in the main text), and the resulting cross-plane thermal diffusivity ($\alpha_z = 0.24 \pm 0.03\ \mathrm{mm^2/s}$) is also similar to that of a previously measured doped sample without a coating ($\alpha_z = 0.25\ \pm 0.03\ \mathrm{mm^2/s}$). Note that in extracting these thermal diffusivities for the coated sample, we did not include the coating in the thermal model, because the thermal diffusion timescale through 1 μm of graphite is at least three orders of magnitude faster than the thermal diffusion timescale through the 20 μm CNT film. Figure S4b shows similar measurements on a graphite-coated CNT film sample for the in-plane y direction measurements. As in the cross-plane case, the in-plane data measured with a coating is similar to the data measured without a coating (see Figure S9b).

Figure S4c shows the cross-plane measurements of a validation 25 μm thick Kapton® polyimide film sample (Toray Industries) with a $\sim$1 μm thickness graphite spray coating on each side. We selected this sample as a validation that the short-pulse laser flash measurements are appropriate for low-diffusivity films with ~20 μm thickness. The fitting result of $\alpha_z = 0.17 \pm 0.02\ \mathrm{mm^2/s}$ is within 25% of typical values reported in the literature.[7] The short-time feature near 0.2 ms is due to laser energy deposition at the top of the sample due to imperfect graphite coating coverage of the transparent polyimide film, and is accounted for in the optical transparency fitting model implemented in the fitting software (red fitting line). We only use this optical transparency fitting model for this polyimide film, and do not use the model for any other measurements in this work.

Representative detector signals corresponding to laser flash measurements on CNT film samples in the x and z directions are shown in Figure 2 of the main text. Figure S9 shows representative

in-plane y-direction CNT film measurements on doped CNT films at two temperatures. As compared to the x-direction measurements in Figure 2d, the peak in the detector signal in Figure S9 occurs at much longer timescales (> 100 ms vs. ~ 40 ms) despite the shorter slit separation distance (3.5 mm vs. 4.5 mm), reflecting the lower thermal diffusivity in the y-direction compared to the x-direction. Both in-plane y measurements in Figure S9 display notable short-time features (i.e., signals that peak near 0 ms but decay over timescales of ~ 20 ms) that are not accounted for in the thermal model. These short-time features could potentially arise due to diffuse reflection of incident laser flash energy that is then absorbed near the central region of the sample, or could arise due to effects of thermal radiation from the heated region where the laser is absorbed to the center region. This radiation-based mechanism is consistent with the observation that the short-time feature appears more prominent at higher temperatures, as seen by comparing Figure S9a and Figure S9b.

Figure S6a shows the results of α_y measurements in which this short-time feature is of minor importance compared to the primary thermal signal associated with the desired heat diffusion between slits. At even higher temperatures (not shown), the fit between the thermal model and the detector signal is poor, and we do not report the results here. Figure S6a shows that the de-doped sample displays notably higher α_y with a stronger T-scaling compared to the doped sample. This doping trend is consistent with the interpretation that the y-direction diffusivity is primarily attributed to partial bundle alignment in the y direction. Future work evaluating the mechanism and developing thermal models could be useful in exploring the y-direction thermal conductivity.

To further validate that the laser flash configurations do not artificially produce anisotropic thermal diffusivity values, we performed measurements on thermally isotropic nickel foils coated with graphite spray on both surfaces. Representative detector signals for the in-plane and cross-plane configurations are shown in Figure S12. The extracted thermal diffusivities of $24.9 \pm 1.2\ \mathrm{mm}^2/\mathrm{s}$ and $18.8 \pm 0.2\ \mathrm{mm}^2/\mathrm{s}$ for the in-plane and cross-plane configurations, respectively, are in good agreement with the literature value of $23\ \mathrm{mm}^2/\mathrm{s}$ for nickel. These results confirm that the measurement geometries and thermal models do not introduce significant artificial anisotropy into the extracted diffusivities.

The adiabatic temperature rise ΔT of a sample during the cross-plane laser flash experiment can be estimated as $\Delta T = E_{\mathrm{pulse}}/Cd$, where E_{pulse} is the absorbed pulse energy density per unit area,

C is the heat capacity per unit volume, and d is the sample thickness. This expression neglects any lateral heat spreading and any losses from the sample. The worst-case scenario temperature rise in the cross-plane measurements occurs for the thin de-doped CNT film at $213\ \mathrm{K}$ with $C = 0.6\ \mathrm{MJ/m^3K}$, $d = 14\ \mu\mathrm{m}$, and $E_{\mathrm{pulse}} = 0.008\ \mathrm{J/cm^2}$, leading to $\Delta T \approx 10\ \mathrm{K}$.

C. Analytical model of along-alignment thermal diffusivity for a hexagonal arrangement of CNTs using a 1D Born-von Karman dispersion relation

We wish to develop a simple analytical model to assess the importance of different phonon scattering mechanisms for the along-alignment CNT conductivity. The model cannot be considered fully quantitative, because of the many simplifying approximations involved in the analysis; our goal is to understand the relevant physics that are at play in determining the thermal properties and to motivate further detailed numerical work exploring phonon thermal transport in complex CNT macrostructures. This one-dimensional model focuses on predicting the x-direction thermal transport with the goal of understanding the $\alpha_x(T)$ trends shown in Figure 3(a). More complex analytical models can be used to understand T-dependent transport along multiple directions in anisotropic materials, but we do not pursue these models here because they require additional fitting parameters, and do not include the inter-bundle contact resistance/porosity effects that likely influence the cross-plane thermal transport.

Following a standard approach for analytical phonon thermal conductivity modeling,[1] we interpret the $\alpha_x(T)$ trends using a simple one-dimensional (1D) phonon transport model prediction for the thermal conductivity

$$\kappa_x = \sum_j C_j v_j \Lambda_j, \qquad (S1)$$

where j is an index labeling the phonon wavevector and polarization, $C_j = \hbar\omega_j \frac{df_{\mathrm{BE}}}{dT}$ is the mode-wise specific heat capacity per unit volume, ω is the phonon frequency, $\hbar$ is Planck's reduced constant, $f_{\mathrm{BE}} = \left(\exp\left(\frac{\hbar\omega}{k_{\mathrm{B}}T}\right) - 1\right)^{-1}$ is the Bose-Einstein distribution, k_{B} is the Boltzmann constant, and v_j and Λ_j are the mode-wise 1D phonon group velocity and 1D mean free path, respectively. The true phonon dispersion relation of CNT materials depends on the diameter and displays van-Hove singularities at the edge of the zone-folded Brillouin zone.[2] For simplicity, we

treat the CNT phonons as a simpler atomic-chain-like 1D system using a triply degenerate sine-type Born-von Karman (BvK) phonon dispersion relation of

$$\omega(q) = \omega_0 \sin\left(\frac{\pi}{2}\frac{q}{q_m}\right) \qquad (S2)$$

where ω is the phonon frequency, q is the phonon wavevector along the CNT axis direction, and ω_0 is the phonon frequency at the maximum wavevector, q_m. For a hexagonally packed array of CNTs with diameter D_{CNT} shown in the inset of Figure S10a, $q_m = \pi\eta_{\text{atoms}}$, where $\eta_{\text{atoms}} = 3.0 \times 10^{11}\ \text{m}^{-1}$ is the number density of atoms per unit length per CNT. We find η_{atoms} by dividing the mass density of annealed, fully-densified, solution-spun CNT fibers ($1710\ \text{kg/m}^3$)[3] by the atomic mass of carbon and multiplying by $\frac{\sqrt{3}}{2}D_{\text{CNT}}^2$, using an estimated value of $D_{\text{CNT}} = 2\ \text{nm}$ for our primarily double-wall CNTs. The phonon density of states per unit length $D(\omega)/L$ for a single polarization in the 1D BvK model is then

$$\frac{D(\omega)}{L} = \frac{1}{\pi v_s}\left(1 - \left(\frac{\omega}{\omega_0}\right)^2\right)^{-1/2}, \qquad (S3)$$

where $v_s = \pi\omega_0/2q_m$ is the speed of sound.

The phonon heat capacity per unit volume is calculated as

$$C = 3\int_0^{\omega_m} \hbar\omega \frac{\partial f_{\text{BE}}}{\partial T}\frac{D(\omega)}{L}\, d\omega, \qquad (S4)$$

where the factor of three arises from the three degenerate phonon polarizations. The thermal diffusivity is then calculated as $\alpha_x = \kappa_x/C_{\text{porous}}$, where C_{porous} is C multiplied by a correction factor $(0.87/1.71)$ to account for the fact that the CNT films are not fully densified.

Figure S10a shows the result of calculating C with Eq. (S4) (solid black line) over the temperature range of 100 K to 2000 K. The constant ω_0 in Eq. (S2) is determined by fitting the analytical model to volumetric heat capacity data of de-doped CNT film (gray squares) obtained from DSC experiments shown in Figure S5. From the single parameter fit, we find that $\omega_0 = 2.4 \times 10^{14}\ \frac{\text{rad}}{\text{s}}$, which corresponds to a maximum phonon frequency of 38 THz and a BvK temperature $T_{\text{BvK}} \equiv \frac{\hbar\omega_0}{k_{\text{B}}} = 1800$ K, both of which have an order-of-magnitude consistency with expectations from more rigorous phonon band structure calculations.[4] The analytical model also exhibits self-

consistency as the high temperature C approaches the Dulong-Petit limit (dashed red line). At temperatures well below T_{BvK}, the specific heat scales linearly with T, in agreement with prior measurements on SWCNT materials for T between 50 K and 300 K.[2]

Converting the sum over modes in Eq. (S1) into an integral over frequency, the thermal conductivity is

$$\kappa_x = 3\int_0^{\omega_m} \hbar\omega \frac{\partial f_{\mathrm{BE}}}{\partial T}\frac{D(\omega)}{L} v(\omega)\Lambda(\omega)d\omega \qquad (S5)$$

where $v(\omega)$ is the phonon group velocity, and $\Lambda(\omega) = \left(\Lambda_{\mathrm{bdy}}^{-1} + \Lambda_I^{-1} + \Lambda_U^{-1}\right)^{-1}$ is the phonon mean free path. Following the Born-von Karman Slack (BvKS) model described in Fang and Dames,[1] $\Lambda_{\mathrm{bdy}} = c_0$ is the (frequency-independent) boundary scattering mean free path, $\Lambda_U^{-1} = c_1 T\omega^2 \exp\left(-\frac{T_{\mathrm{BvK}}}{2T}\right) v_g^{-1}$ is the inverse of the Umklapp mean free path (including the exponential term arising from phonon freezeout at T well below T_{BvK}), and $\Lambda_I^{-1} = c_3\omega^4 v_g^{-1}$ is the inverse of the impurity scattering mean free path.

Figure S10b and Figure 3d in the main text show the analytical calculation of $\kappa_x(T)$ and $\alpha_x(T)$ with and without impurity scattering (dashed black line and solid black line, respectively) over the temperature range 100 K to 2000 K. To determine the fitting parameters c_0 and c_1, we performed a two-parameter fit in which the result from Eq. (S5), excluding impurity scattering, was fitted to in-plane thermal diffusivity data of de-doped CNT films obtained via the laser flash method (discussed in the main text). As seen in Figure 3d, values of $\Lambda_{\mathrm{bdy}} = 0.6\ \mu\mathrm{m}$ and $c_1 = 1.6 \times 10^{-21}\ \mathrm{s\ K^{-1}}$ produce close agreement between the impurity-free model (solid black curve) and the measured α_x of the de-doped CNT film (orange circles) across the full temperature range that we investigated. To determine c_3, we performed a single parameter fit in which the result from Eq. (S5), including impurity scattering (dashed black curve), was fitted to experimentally determined α_x of doped CNT films (blue circles); the value of $c_3 = 9 \times 10^{-47}\ \mathrm{s}^3$ gives the best agreement with the data.

While this kinetic theory thermal transport model is simple and neglects many realistic features of phonon transport in solution-spun CNT films, the model does demonstrate the origins of the negative temperature dependence seen in α, and enables first estimation of the relevant phonon

scattering mechanisms. The model predicts that even larger α_x could be obtained if larger boundary scattering mean free paths were possible; for example, Figure 3d shows that if $\Lambda_{\mathrm{bdy}} =$ 2.5 μm, α_x without impurity scattering exceeds $1000\ \mathrm{mm}^2/\mathrm{s}$ near 300 K and exhibits a maximum temperature scaling that is similar to T^{-2} (i.e., a result that is qualitatively similar to the experimental finding of the HOPG sample). This kinetic theory model of α_x shows that strong T scalings of α_x (similar to or even stronger than T^{-1}) can be observed below the BvK temperature in CNT materials, motivating further investigation of phonon scattering mechanisms and strategies to enhance the phonon mean free path.

D. Characteristic time scale associated with parasitic heat losses for the in-plane laser flash configuration

In-plane laser flash measurements must contend with potential parasitic heat losses due to convection or radiation. Given that our laser flash measurements were conducted in air, natural convection was the dominant heat-loss mechanism such that the characteristic time for losses to the surroundings is

$$t_{\mathrm{loss}} = \frac{\rho c_p d}{h_{\mathrm{conv}}}, \qquad (S6)$$

where ρ is the mass density of the sample, c_p is the specific heat capacity of the sample, and h_{conv} is the heat transfer coefficient due to natural convection. To estimate h_{conv}, we used the Nusselt number (Nu) definition,

$$\mathrm{Nu} = h_{\mathrm{conv}} \frac{w}{\kappa_{\mathrm{air}}}, \qquad (S7)$$

where w is the width of the sample (which is the characteristic heat transfer length in this context) and κ_{air} is the thermal conductivity of the surrounding air. For natural convection from an upward facing heated surface of width w, Nu is related to the Rayleigh number (Ra), defined as

$$\mathrm{Ra} = \frac{g\beta(T_s - T_\infty)w^3}{\nu \alpha_f}, \qquad (S8)$$

where g is the gravitational acceleration, β is the thermal expansion coefficient of the surrounding fluid, T_s is the surface temperature of the sample, T_∞ is the far-field fluid temperature, ν is the kinematic viscosity of the surrounding fluid, and α_f is the thermal diffusivity of the surrounding

fluid. For our typical temperature rises and geometries, we find that $\mathrm{Ra} \sim 1$, meaning that the effects of fluid motion are minimal and the order-of-magnitude of the convection coefficient h_{conv} can be estimated by setting $\mathrm{Nu} = 1$ in Eq. (S7). Doing so results in $h_{\mathrm{conv}} \approx 30\ \mathrm{W/m^2K}$ per film surface, which we conservatively round up to $h_{\mathrm{conv}} = 100\ \mathrm{W/m^2K}$ as a worst-case scenario for the effective heat transfer coefficient for the whole sample.

From the samples we measured, the smallest value of t_{loss} was for a doped CNT film: $d = 14$ µm, $\rho = 1300\ \mathrm{kg/m^3}$, $c_p = 740\ \mathrm{J/kg.K}$. Plugging in $h_{\mathrm{conv}} = 100\ \mathrm{W/m^2K}$ and the relevant sample properties into Eq. (S6) resulted in $t_{\mathrm{loss}} \approx 150\ \mathrm{ms}$. For all other CNT films, t_{loss} was $150 - 200\ \mathrm{ms}$, and for HOPG, $t_{\mathrm{loss}} \approx 1\ \mathrm{s}$. These times are all much longer than the measured in-plane along-alignment diffusion timescales shown in Figure 4b, confirming that losses are negligible. This timescale is also longer than the in-plane y direction timescale shown in Figure 4b, though the slower time response for the α_y measurements leads to time constants that are approaching the loss-limited values. The algebraic relation $t_{1/2} = 0.1\, L^2/\alpha$, used to generate the contours in Figure 4b of the main text, comes from the criteria that $t_{\mathrm{loss}} \geq 10\ t_{1/2}$.

E. Heat spreading anisotropy of a simple polymer-metal foil composite

This work focused on intrinsic anisotropy in a single CNT film. For thermal applications, it is also possible to achieve anisotropic heat spreading using a composite film, such as a metal foil with a polymer coating. We wish to compare the performance of the single-material anisotropic system with the two-material composite.

Consider materials 1 and 2, each of which have an isotropic thermal conductivity κ_1 and κ_2 and thickness d_1 and d_2 in the z-direction. If there is no thermal contact resistance between the materials, the effective thermal conductivity for heat transfer in the cross-plane direction $\kappa_{\mathrm{eff,cp}}$ is

$$\kappa_{\mathrm{eff,cp}} = \left(\frac{\gamma}{\kappa_1} + \frac{(1-\gamma)}{\kappa_2}\right)^{-1}, \qquad (S9)$$

where $\gamma = d_1/(d_1 + d_2)$ is a dimensionless thickness ratio. The effective thermal conductivity in the in-plane direction is

$$\kappa_{\mathrm{eff,ip}} = \kappa_1\gamma + \kappa_2(1-\gamma). \qquad (S10)$$

For all κ_1 and κ_2, the maximum effective thermal conductivity anisotropy $\kappa_{\text{eff,ip}}/\kappa_{\text{eff,cp}}$ is obtained for $\gamma = 0.5$, and has the value

$$\left(\frac{\kappa_{\text{eff,ip}}}{\kappa_{\text{eff,cp}}}\right)_{\text{max}} = \frac{1}{4}\left(\frac{\kappa_1}{\kappa_2}\right)\left(1 + \frac{\kappa_2}{\kappa_1}\right)^2. \qquad (S11)$$

Without loss of generality, assume $\kappa_1 > \kappa_2$. The best possible anisotropy ratio of the composite film approaches $\frac{1}{4}\left(\frac{\kappa_1}{\kappa_2}\right)$ when $\kappa_1 \gg \kappa_2$; the factor of ¼ reflects the fact that only half of the composite film thickness acts to block cross-plane heat flow, while only half of the composite film thickness enables in-plane heat flow. Therefore, even a combination of metal foil (e.g. $\kappa_1 = 400\ \text{W/mK}$ for copper) and a polymer coating (e.g. $\kappa_2 = 0.2$ W/m.K for polyimide) can only achieve an anisotropy ratio of $\frac{1}{4}\left(\frac{\kappa_1}{\kappa_2}\right) = 500$, which is similar to that of graphite and well below that of the CNT film. Higher effective thermal anisotropy can be achieved by using materials with higher thermal conductivity contrast or by maintaining gaps between material 1 and 2 that block the cross-plane heat flow but enable in-plane heat flow. Further design of such multi-material heat spreading systems could be an alternate method for vertical thermal isolation in electronic applications.

F. Specific heat capacity measurements of CNT films using differential scanning calorimetry

We measured the temperature-dependent specific heat capacity (c_p) of solution-spun CNT films using differential scanning calorimetry (NETZSCH differential scanning calorimetry (DSC) 3500 Sirius). We performed all DSC measurements using a platinum (Pt) pan heat-capacity protocol with sapphire as the reference material. For each set of measurements, we acquired baseline, sapphire reference, and sample scans sequentially on the same day to reduce systematic error. We cut solution-spun CNT films into small pieces and placed them into Pt crucibles. We weighed each sample prior to measurement using a microbalance. The doped CNT film had a mass of 8.4 mg, and the de-doped CNT film had a mass of 8.1 mg. The Pt pans had a nominal mass of approximately 25 mg. We first acquired baseline measurements using two empty Pt pans. We then placed a sapphire standard in the reference pan and performed the reference measurement to determine instrument sensitivity. After completing the sapphire measurement, we removed the

reference and placed the CNT film sample into the Pt pan. We conducted all measurements under a nitrogen purge to suppress oxidation and minimize moisture effects. Before each measurement, we ran a smart cleaning and heating cycle to remove residual water vapor from the DSC chamber, followed by controlled cooling to the measurement start temperature. We initiated measurements only after the system temperature stabilized at 25 ± 5 °C.

G. Electrical resistance measurements on CNT films

The in-plane electrical conductivity of doped and de-doped solution-spun CNT films was measured using a four-probe configuration adapted from prior measurements on CNT fibers.[5] Rectangular strips were cut such that the applied current and measured voltage drop were oriented either parallel (x-direction) or perpendicular (y-direction) to the CNT alignment. The strip length (distance between voltage probes) ranged from 3.55 to 27 mm depending on measurement direction, while the strip width ranged from 0.51 to 1.35 mm. The film thickness was measured independently and used to define the cross-sectional area assuming a uniform thickness across the strip. Electrical contacts were formed using conductive silver paint (Ted Pella) and 50 μm diameter silver wire leads (Goodfellow). Electrical resistance was obtained from linear fits to current–voltage (I–V) sweeps performed using a DC current source (Keithley 2400 SourceMeter). The in-plane electrical conductivity was calculated using the inner edge distance between the voltage probes. The reported uncertainty (one standard error) in the electrical conductivity was determined by propagating uncertainties in the cross-sectional area, with the dominant contribution arising from uncertainty in the film thickness.

H. Mechanical compression of CNT films

To investigate the effect of densification on the cross-plane electrical transport properties, we mechanically compressed doped and de-doped CNT films using a Carver 12-ton hydraulic press (Model 3912). We first cut the CNT films into approximately 1 cm × 1 cm pieces using a razor blade. We then sandwiched each CNT film sample between two glass microscope slides (Fisherbrand Plain Glass Microscope Slides, Fisher Scientific) to provide flat and rigid compression surfaces and to reduce handling damage during pressing. Next, we placed the slide-sample-slide assembly between two aluminum support plates inside the hydraulic press. We applied a compressive load of 6 metric tons to the sample and immediately released the load after

reaching the target force without a prolonged holding step. The compression reduced the film thickness by approximately 20% while producing negligible changes in the in-plane sample dimensions. After compression, we applied acetone at the glass-slide interface to release the CNT film from the slides. Finally, we allowed the compressed films to dry under ambient laboratory conditions prior to characterization and laser flash analysis (Figure S13).

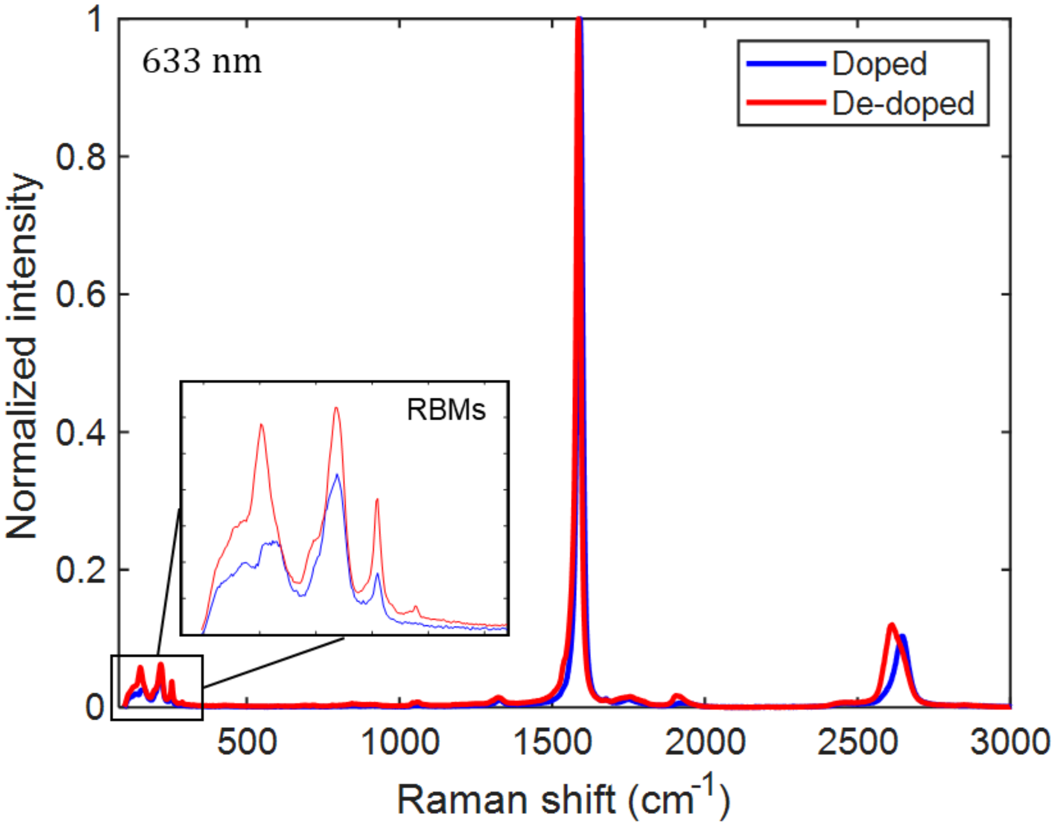

Figure S1. Raman spectra of doped (blue) and de-doped (red) solution-spun CNT films obtained with a 633 nm laser excitation, incident light polarization aligned parallel to the x-direction of the CNT film, and no polarizer used on the detector. The de-doped film was annealed at 800 ℃ for 6 hours in nitrogen gas. Data is normalized to the G peak intensity. Both doped and de-doped samples exhibit large G/D ratios (> 50) and characteristic low-frequency radial breathing modes (inset) associated with the CNT structure. We determine the G/D ratio by subtracting a noise floor associated with the minimum intensity in the Raman spectra and then taking the peak intensity ratio.

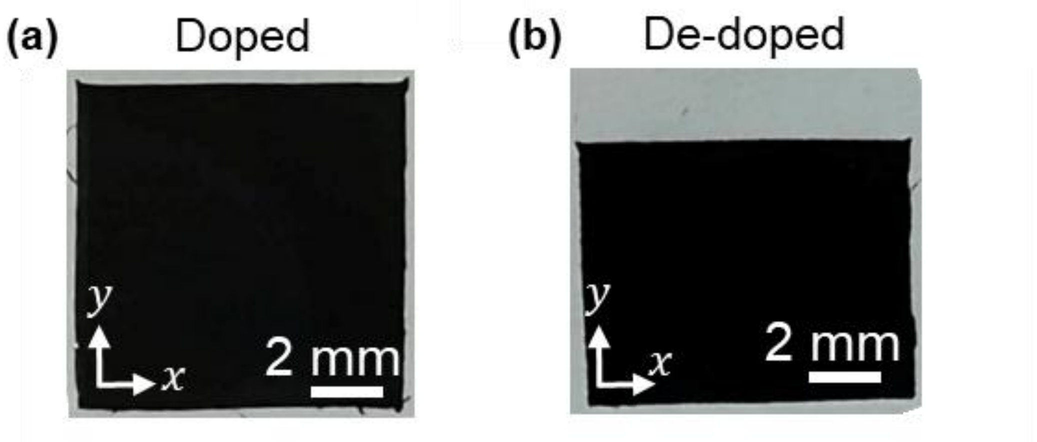

Figure S2. Top-down optical images of a CNT film sample before (a) and after (b) annealing. Substantial shrinkage occurs in the y-direction perpendicular to the CNT alignment. The dimensions and mass of the doped sample are $22\ \mu m \times 8.8$ mm $\times$ 8.9 mm (z, y, x) and 2.23 mg, respectively. The dimensions and mass of the de-doped sample are $22\ \mu m \times 7.1$ mm $\times$ 8.8 mm (z, y, x) and 1.17 mg, respectively. The mass loss is due to removal of the residual CSA derivatives upon annealing.

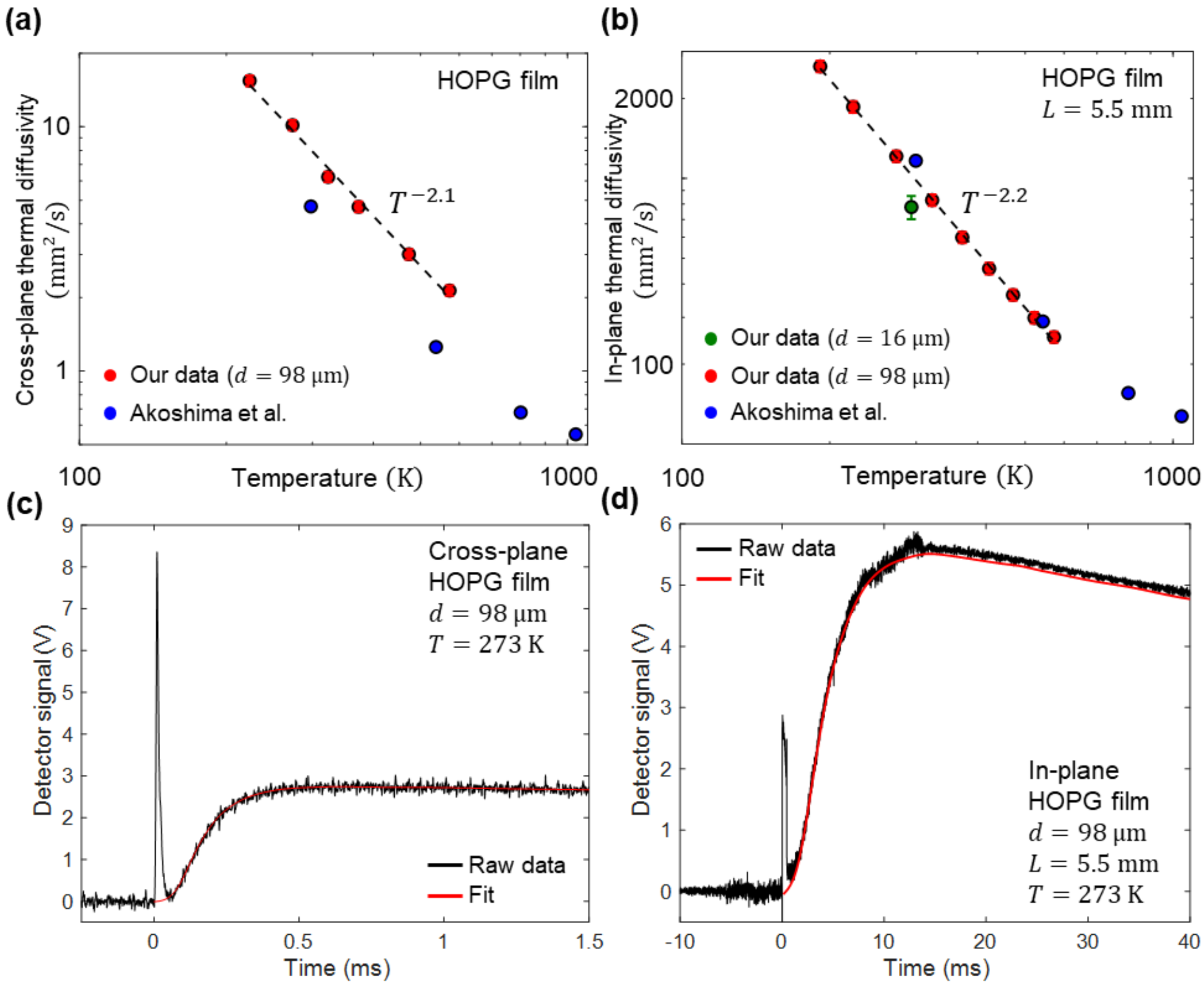

Figure S3. Validation measurements of the laser flash method on highly oriented pyrolytic graphite (HOPG). Temperature dependent (a) cross-plane thermal diffusivity α_z and (b) in-plane thermal diffusivity α_x of a 98 μm thick sample. The temperature dependencies of our measured diffusivities for α_z and α_x are $T^{-2.1}$ and $T^{-2.2}$, respectively. The measured cross-plane diffusivity is slightly larger than the reference data,[6] while the agreement with the in-plane reference data is excellent. We also performed a room-temperature in-plane measurement on a 16 μm thick HOPG film (green), which is within 20% of the value extrapolated from the 98 μm sample from the same vendor. (c) Time-dependent voltage signal from the IR detector for the cross-plane configuration and (d) in-plane configuration, both for a 98 μm thick HOPG sample at 273 K. Black solid lines represent the measured detector signal, while solid red lines correspond to the fitting models used to extract α.

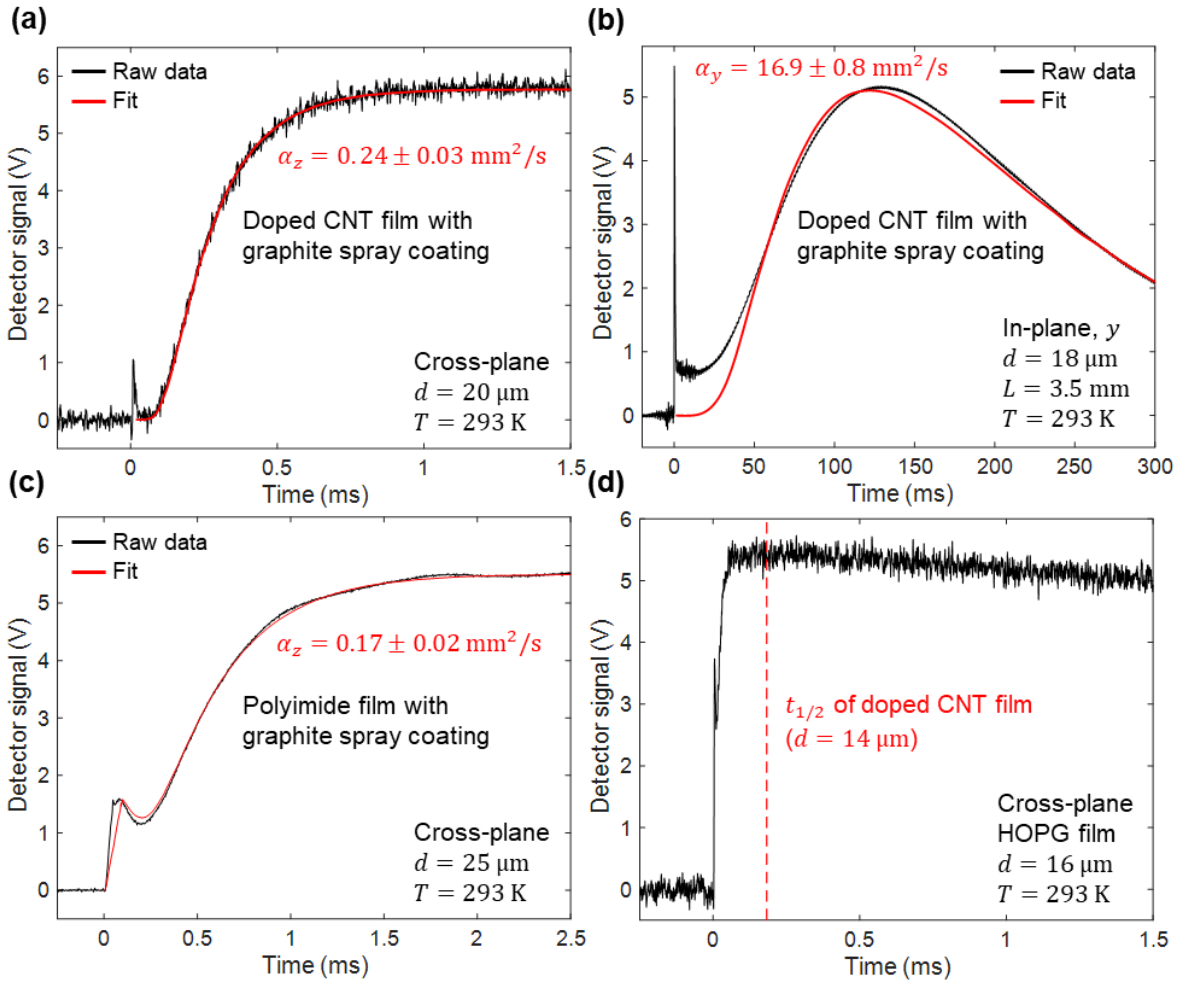

Figure S4. Infrared detector signals as a function of time for validation measurements on ~20 μm films with ~1 μm of graphite spray coating on both surfaces at room temperature. Detector signal profiles for doped CNT films with measurements performed in the (a) cross-plane z direction and (b) in-plane y direction both show similar responses as the samples without coatings (not shown here), showing that the laser heating penetration depth in the CNT film is smaller than the film thickness. (c) Validation measurements on a graphite-coated low-thermal diffusivity 25 μm thick polyimide film yield $\alpha_z = 0.17 \pm 0.02\ \mathrm{mm^2/s}$, which is within 25% of typical values reported in the literature.[7] (d) Detector signal for a 16 μm thick HOPG film acquired at the minimum pulse-width. The rapid detector rise time confirms that the time resolution of the infrared detector is sufficient to resolve the shortest $t_{1/2}$ observed in our CNT film measurements (dashed red line). Black solid lines represent the measured detector signal, while solid red lines in panels (a–c) correspond to the fitting models used to extract α.

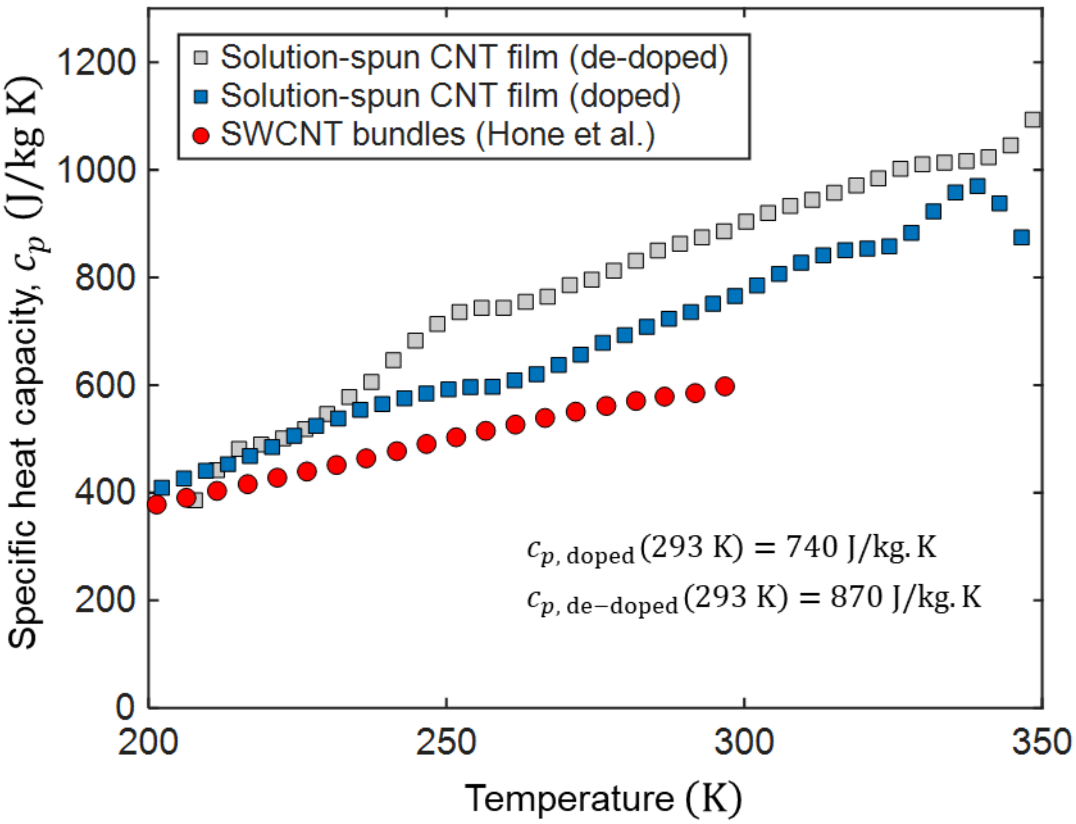

Figure S5. Specific heat capacity (c_p) as a function of temperature for solution-spun CNT film obtained using differential scanning calorimetry (DSC): Blue square markers correspond to doped film, and gray square markers correspond to de-doped film. For reference, the specific heat capacity of a sample consisting of bundles of single-wall CNTs[8] is included over a similar temperature range (red circular markers). Differences in c_p between the CNT film and SWCNT bundles could be due to the CNT wall number and/or sample purity.

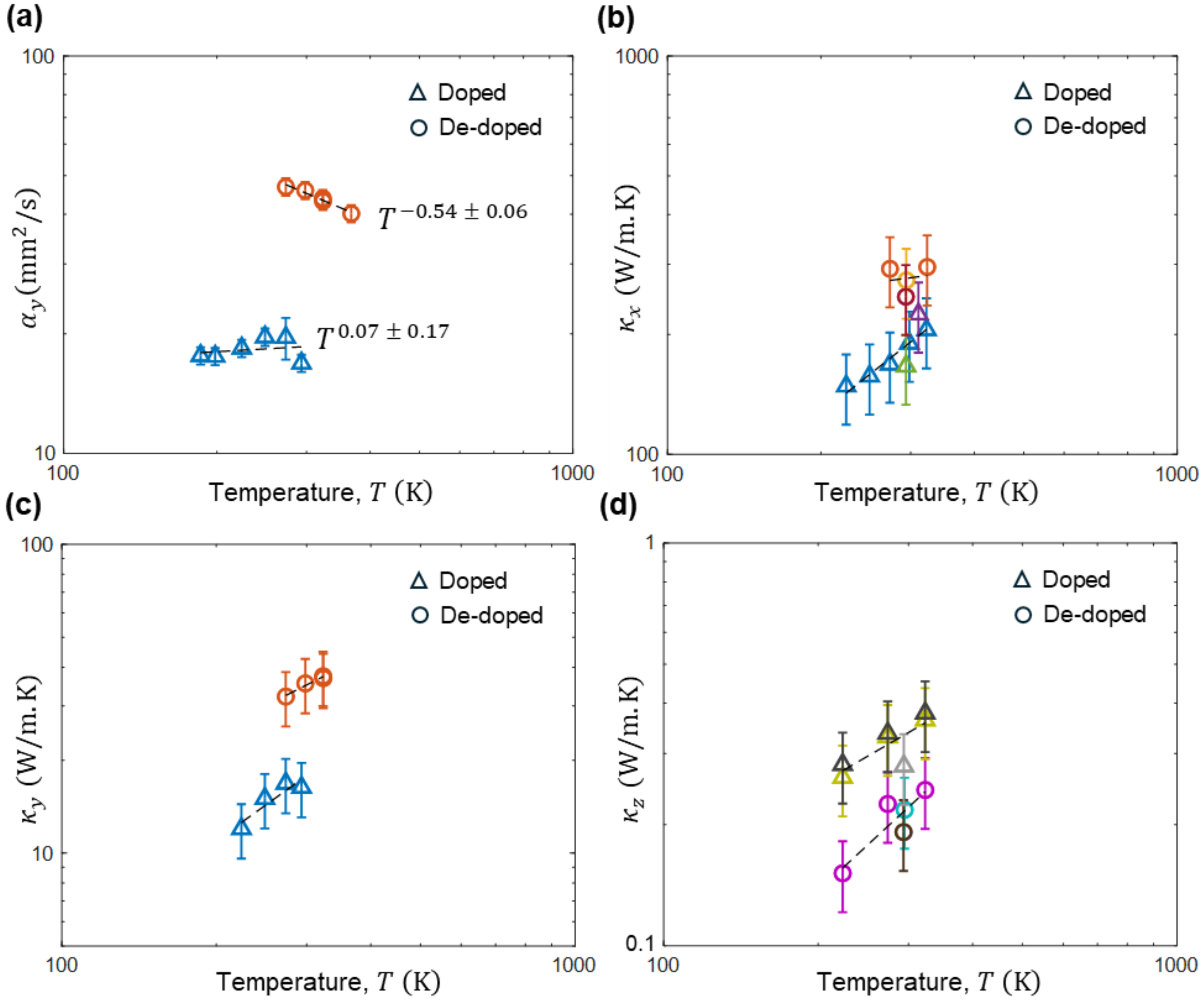

Figure S6. (a) Temperature dependence of in-plane thermal diffusivity perpendicular to the alignment direction (α_y) in doped (triangles) and de-doped (circles) CNT films measured using the laser flash method. (b) Temperature dependence of in-plane x, (c) in-plane y, and (d) and cross-plane thermal conductivities of solution-spun CNT films extracted from a combination of laser flash α measurements and differential scanning calorimetry heat capacity measurements, over the T range where C measurements were performed. Colors represent measurements on different samples and correspond to the same samples shown in Figure 3 of the main text. Dashed lines represent power law fits. Error bars represent the standard error with a 68% confidence interval.

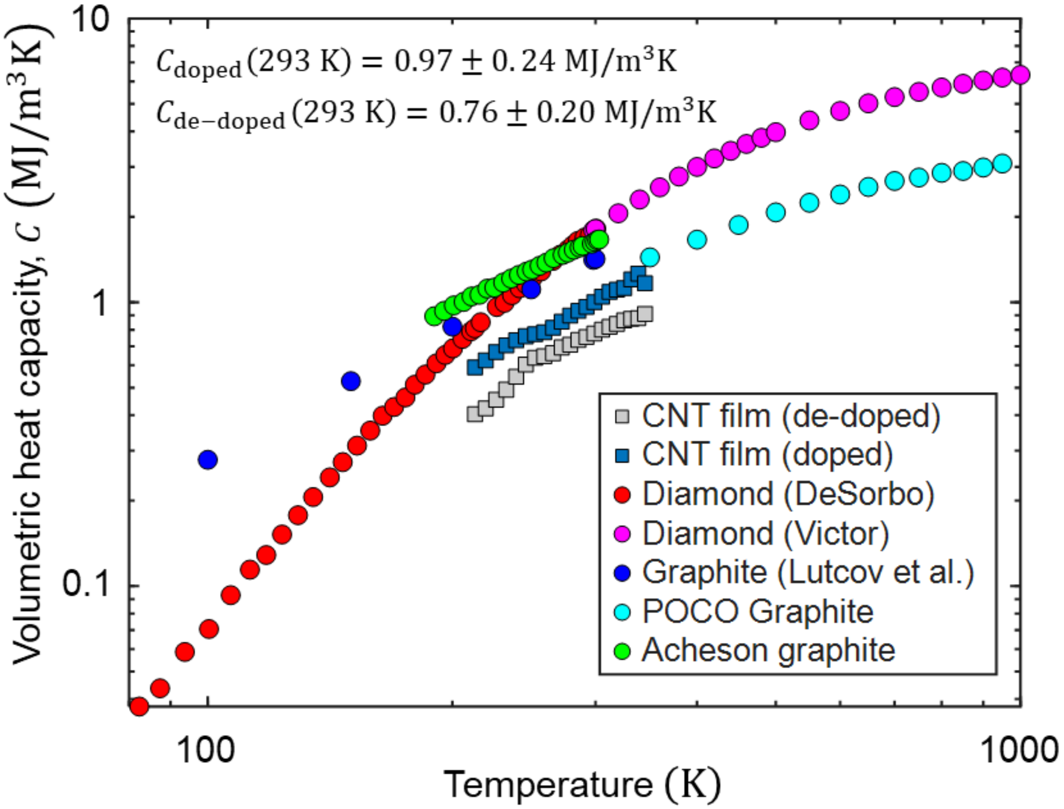

Figure S7. Temperature dependent volumetric heat capacity (C) for solution spun CNT films, diamond,[9,10] and graphite.[11–13] The C of solution-spun CNT films was calculated using the c_p curves in Figure S5 and mass density measurements (doped: $1300\ \mathrm{kg/m^3}$; de-doped: $870\ \mathrm{kg/m^3}$).

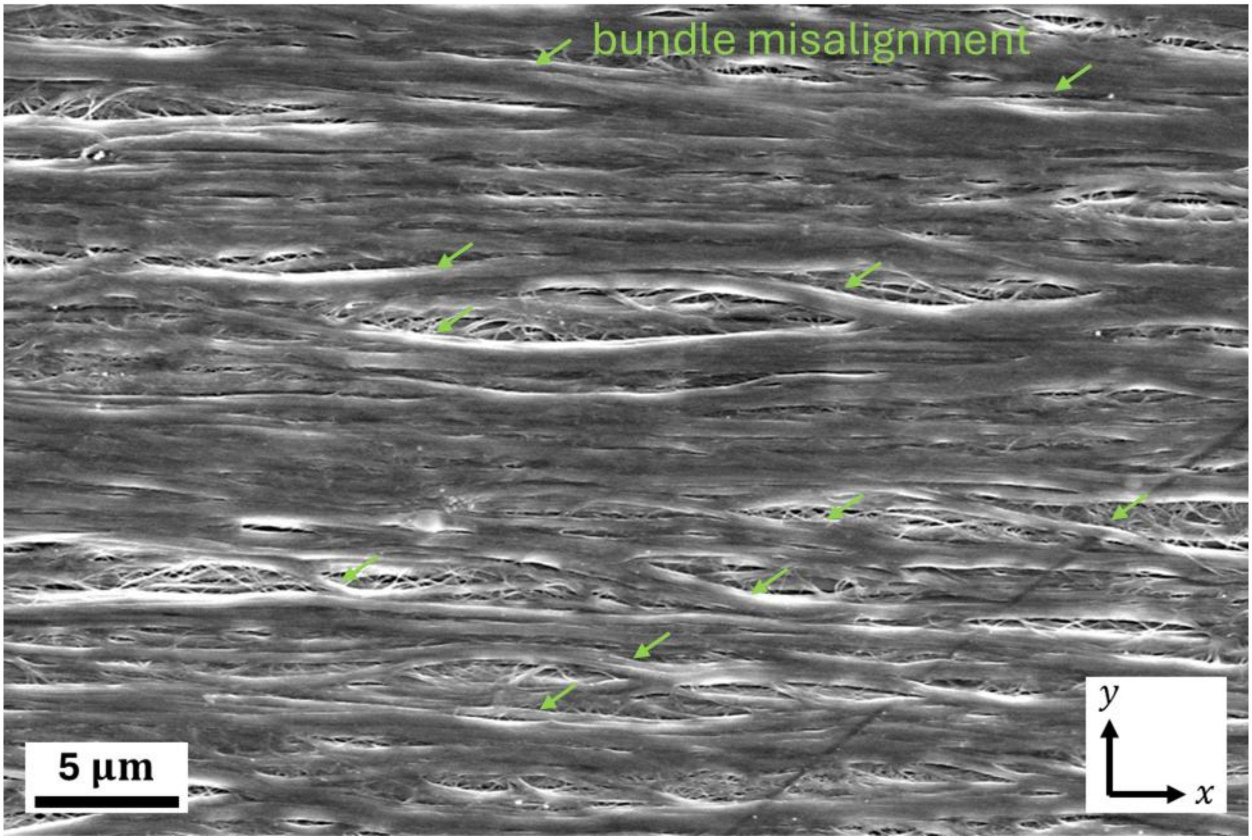

Figure S8. Scanning electron microscope (SEM) top-view image of a de-doped CNT film showing partial orientation of the CNT bundles along the y-direction (green arrows), further supporting the observation in Figure 1c.

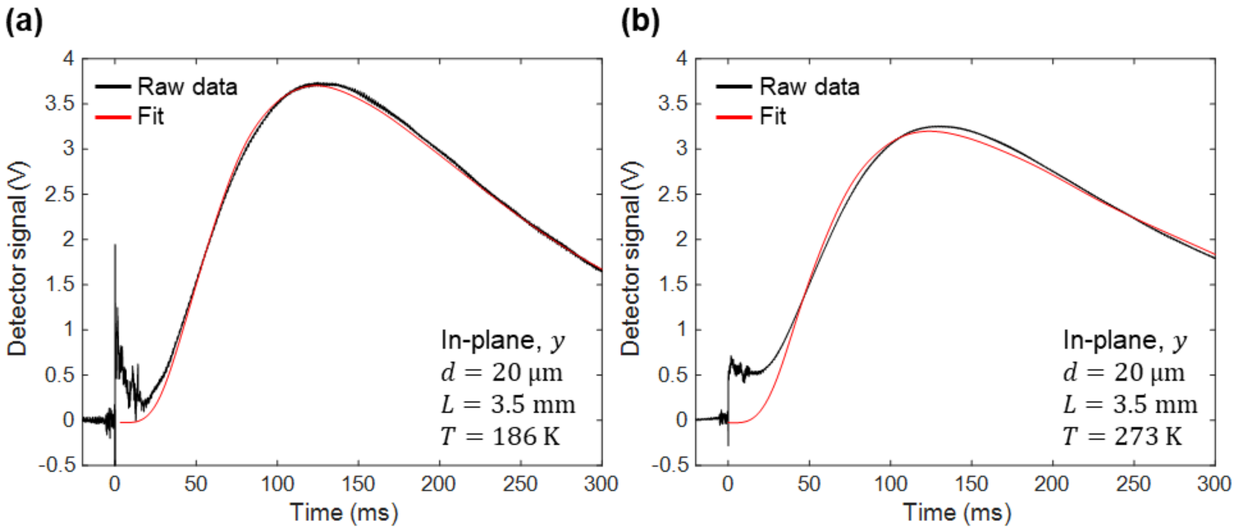

Figure S9. Representative detector voltage signals from in-plane y-direction laser flash measurements on a doped CNT film at (a) 187 K and (b) 273 K. No coatings are applied in these measurements.

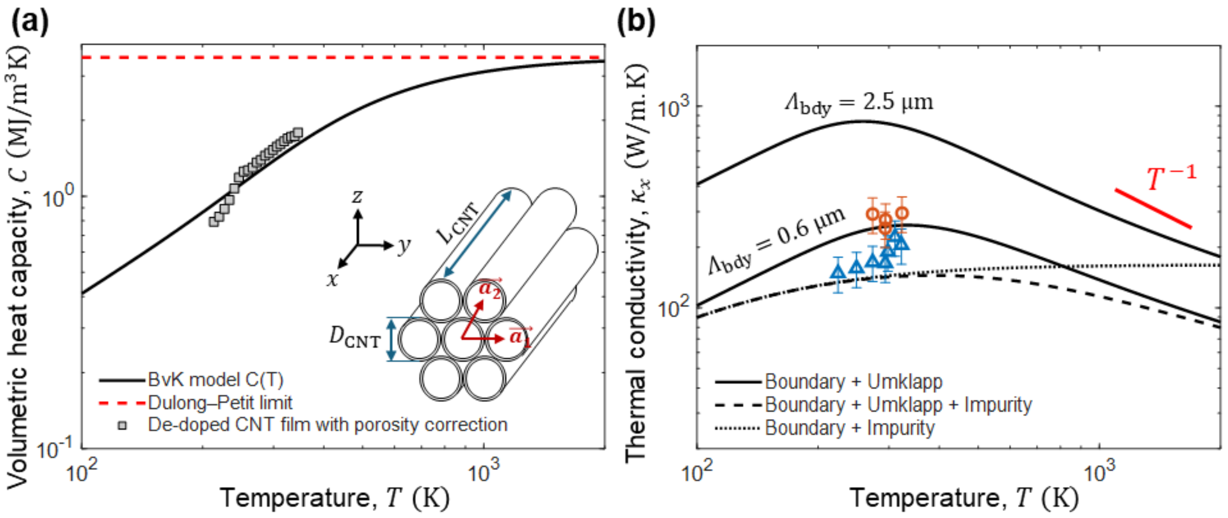

Figure S10. (a) Schematic of a primitive unit cell (defined by vectors $\overrightarrow{a_1}$ and $\overrightarrow{a_2}$) in a hexagonal arrangement of CNTs with an outer diameter of D_{CNT}. The primitive unit cell parameters are used in an analytical model with a 1D Born-von Karman (BvK) dispersion relation and four free-fitting parameters to calculate the volumetric heat capacity C, (b) along-alignment thermal conductivity κ_x, and along-alignment thermal diffusivity α_x (not shown here). $C(T)$ of the de-doped CNT films (gray squares) is used to determine a single fitting parameter in the BvK dispersion relation. $\alpha_x(T)$ of de-doped CNT films (orange circles) is used to determine two fitting parameters within the boundary scattering and Umklapp scattering effective phonon mean free path (MFP) model. α_x of doped CNT films (blue circles) is used to determine a single fitting parameter in the boundary scattering, Umklapp scattering, and impurity scattering effective phonon MFP model.

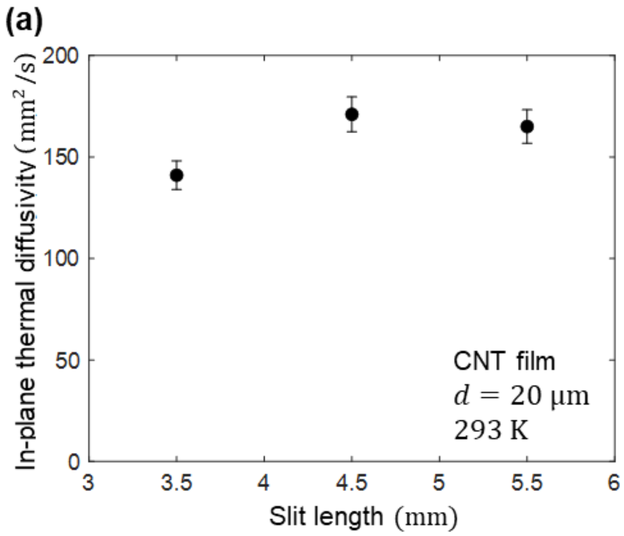

Figure S11. Room temperature in-plane thermal diffusivity of CNT films as a function of slit length separation (L) for CNT films with (a) 20 μm and (b) 14 μm thickness. The extracted diffusivity is similar for different slit lengths, validating the laser flash method.

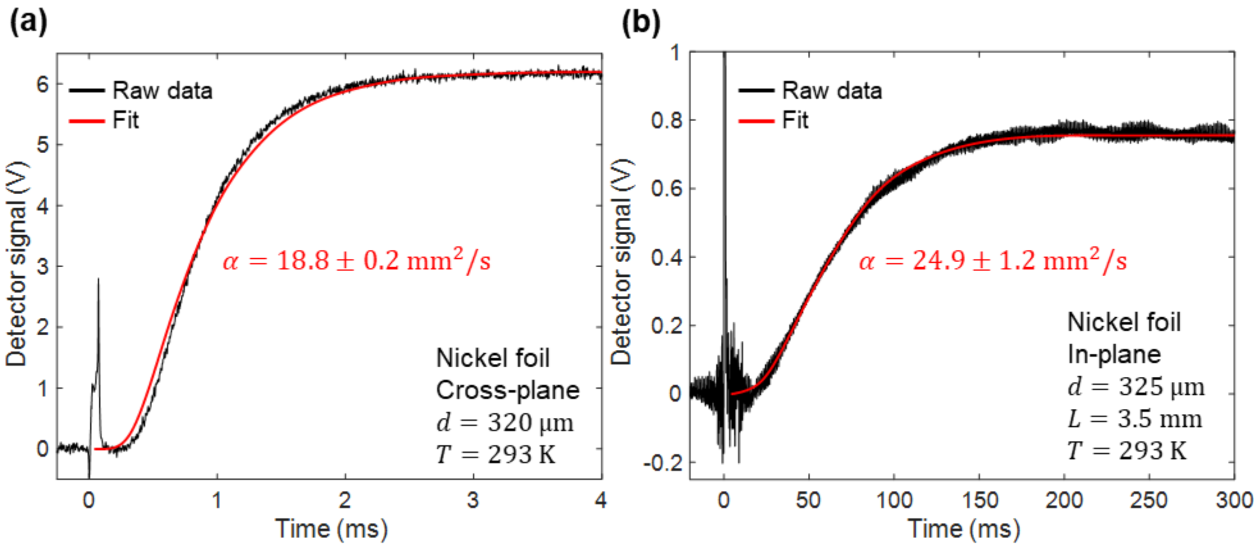

Figure S12. Representative detector voltage signals from laser flash measurements on graphite-coated thermally isotropic nickel foils in the (a) in-plane and (b) cross-plane directions at room temperature. The measured thermal diffusivity values of 24.9 ± 1.2 mm^2/s and 18.8 ± 0.2 mm^2/s for the in-plane and cross-plane measurements, respectively, are in good agreement with the literature value of 23 mm^2/s.

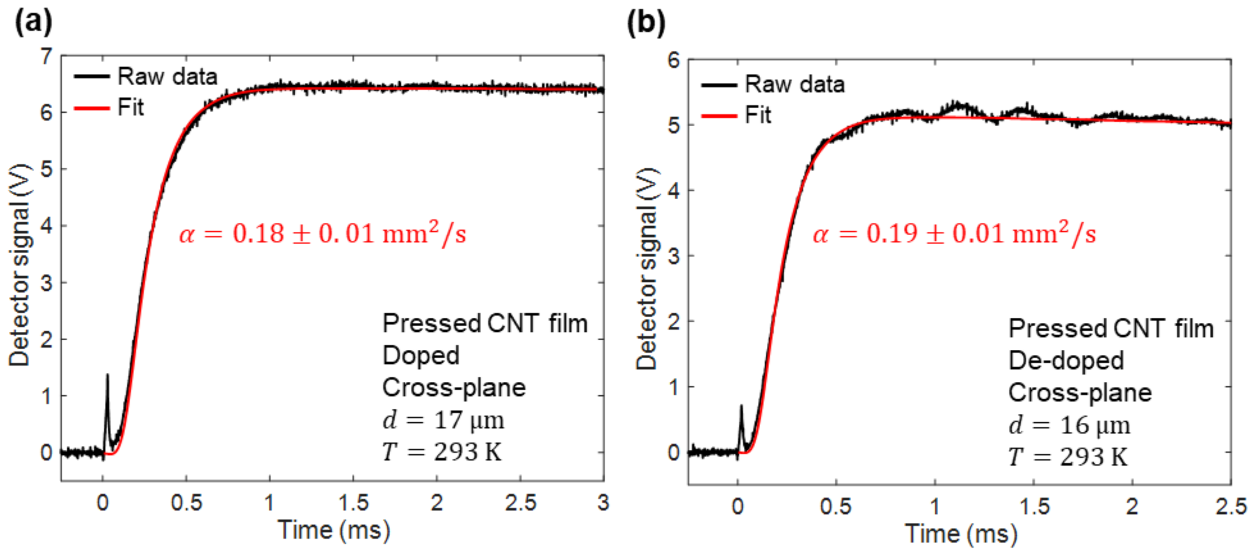

Figure S13. Detector voltage signals from cross-plane laser flash measurements on mechanically compressed CNT films at room temperature. We applied compressive stress to the films as described in Supplementary Note H, resulting in a ~20% reduction in film thickness. Representative detector signals are shown for compressed (a) doped and (b) de-doped CNT films. These films have lower α_z than their non-compressed CNT films, due to their higher volumetric heat capacity and similar κ_z.

TABLE S1. In-plane electrical conductivities of solution-spun CNT film at room temperature.

Sample condition	σ_x (MS/m)	σ_y (MS/m)
Doped	5.4 ± 0.3	0.23 ± 0.01
De-doped	1.5 ± 0.08	0.14 ± 0.01

SUPPORTING INFORMATION REFERENCES

(1) Yang, F.; Dames, C. Mean Free Path Spectra as a Tool to Understand Thermal Conductivity in Bulk and Nanostructures. *Phys. Rev. B* **2013**, *87* (3), 035437. https://doi.org/10.1103/PhysRevB.87.035437.

(2) Hone, J.; Batlogg, B.; Benes, Z.; Johnson, A. T.; Fischer, J. E. Quantized Phonon Spectrum of Single-Wall Carbon Nanotubes. *Science* **2000**, *289* (5485), 1730–1733. https://doi.org/10.1126/science.289.5485.1730.

(3) Heo, S. J.; Kim, J.; Choi, G. M.; Lee, D.; Im, B. W.; Kim, S.-S.; Ku, B.-C.; Lee, H. S.; Kim, S. G. Microstructural Evolution Effects on the Density of Carbon Nanotube Fibers. *Carbon* **2024**, *226*, 119180. https://doi.org/10.1016/j.carbon.2024.119180.

(4) Dubay, O.; Kresse, G. Accurate Density Functional Calculations for the Phonon Dispersion Relations of Graphite Layer and Carbon Nanotubes. *Phys. Rev. B* **2003**, *67* (3), 035401. https://doi.org/10.1103/PhysRevB.67.035401.

(5) Song, Y.; Durán-Chaves, M.; Siqueira, I. R.; Dewey, O. S.; Stefanov, O.; Komatsu, N.; Kono, J.; Pasquali, M.; Wehmeyer, G. Molecular Aspect Ratio Effect on Axial Thermal Transport in Solution-Spun Carbon Nanotube Fibers. *J. Appl. Phys.* **2025**, *137* (10), 105101. https://doi.org/10.1063/5.0244895.

(6) Akoshima, M.; Abe, H.; Baba, T. Thermal Diffusivity of Carbon Materials as Candidate Reference Materials. *Int. J. Thermophys.* **2015**, *36* (10), 2507–2517. https://doi.org/10.1007/s10765-014-1624-2.

(7) Morikawa, J.; Hashimoto, T. Thermal Diffusivity of Aromatic Polyimide Thin Films by Temperature Wave Analysis. *J. Appl. Phys.* **2009**, *105* (11). https://doi.org/10.1063/1.3116509.

(8) Hone, J.; Llaguno, M. C.; Biercuk, M. J.; Johnson, A. T.; Batlogg, B.; Benes, Z.; Fischer, J. E. Thermal Properties of Carbon Nanotubes and Nanotube-Based Materials. *Appl. Phys. A* **2002**, *74* (3), 339–343. https://doi.org/10.1007/s003390201277.

(9) DeSorbo, W. Specific Heat of Diamond at Low Temperatures. *J. Chem. Phys.* **1953**, *21* (5), 876–880. https://doi.org/10.1063/1.1699050.

(10) Victor, A. C. Heat Capacity of Diamond at High Temperatures. *J. Chem. Phys.* **1962**, *36* (7), 1903–1911. https://doi.org/10.1063/1.1701288.

(11) Lutcov, A. I.; Volga, V. I.; Dymov, B. K. Thermal Conductivity, Electric Resistivity and Specific Heat of Dense Graphites. *Carbon* **1970**, *8* (6), 753–760. https://doi.org/10.1016/0008-6223(70)90100-4.

(12) Taylor, R. E.; Groot, H. Thermophysical Properties of POCO Graphite; PRL-153, Interim Report; Properties Research Laboratory, Purdue University: West Lafayette, IN, 1978.

(13) DeSorbo, W.; Tyler, W. W. The Specific Heat of Graphite from 13° to 300°K. *J. Chem. Phys.* **1953**, *21* (10), 1660–1663. https://doi.org/10.1063/1.1698640.
